# Supplementary material for: Transcriptional Convergence of Oligodendrocyte Lineage Progenitors during Development
Source: Dev Cell. 2018 Aug 20;46(4):504–517.e7. doi: 10.1016/j.devcel.2018.07.005 (PMC6104814; doi:10.1016/j.devcel.2018.07.005)
Supplement: Document S1. Figures S1–S6 [file mmc1.pdf]

Developmental Cell, Volume *46*

Supplemental Information

Transcriptional Convergence of Oligodendrocyte Lineage Progenitors during Development

Sueli Marques, David van Bruggen, Darya Pavlovna Vanichkina, Elisa Mariagrazia Floriddia, Hermany Munguba, Leif Väremo, Stefania Giacomello, Ana Mendanha Falcão, Mandy Meijer, Åsa Kristina Björklund, Jens Hjerling-Leffler, Ryan James Taft, and Gonçalo Castelo-Branco

a

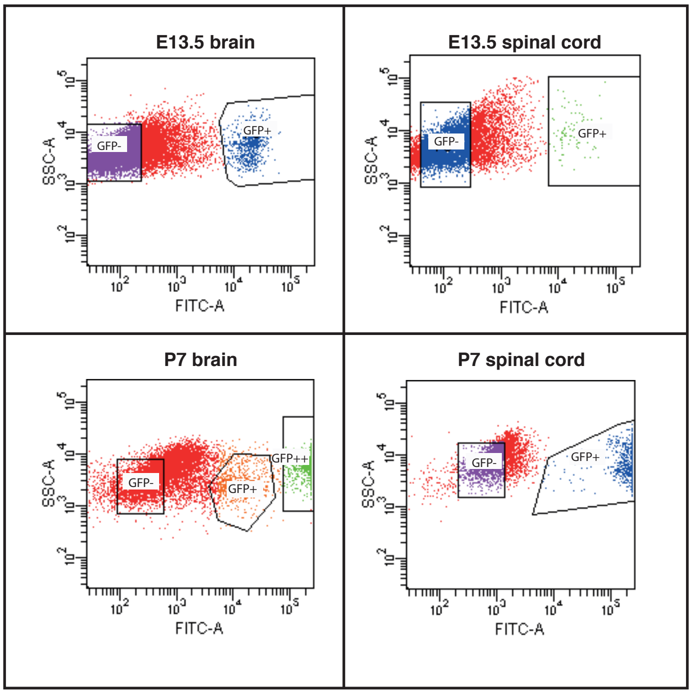

b

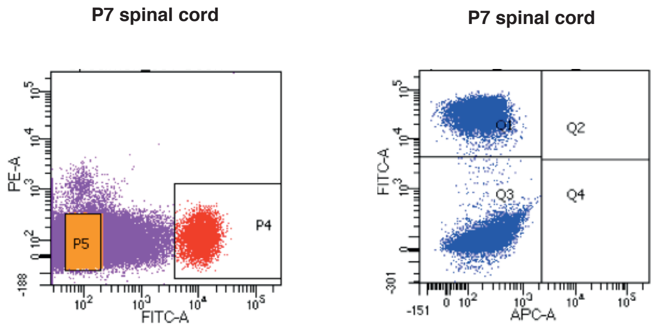

c

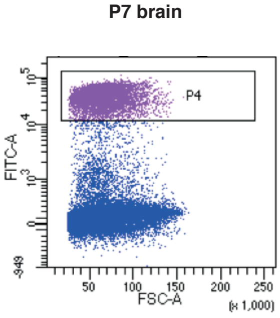

d

Dataset	Reads	Uniquely mapped	Median TIN	Spliced Reads	Stranding	Partially novel SJ	Novel SJ	Total SJ	GTAG	GCAG	ATAC	Non-canonical SJ
**E13.5B1**	46837623	87.73%	76.78	24.03%	98.87%	16%	8%	13424516	98.99%	0.86%	0.10%	0.06%
**E13.5B2**	53041658	86.42%	77.01	22.33%	98.08%	15%	8%	13995601	98.98%	0.86%	0.10%	0.06%
**E13.5B3**	42836948	83.84%	74.90	23.78%	98.29%	14%	8%	12104537	98.97%	0.87%	0.10%	0.07%
**E13.5S1**	46481407	88.30%	75.49	17.43%	98.77%	14%	8%	9710372	99.05%	0.80%	0.09%	0.06%
**E13.5S2**	60703882	89.39%	76.32	17.59%	97.63%	15%	9%	12791166	99.05%	0.80%	0.09%	0.06%
**E13.5S3**	37869824	72.44%	67.54	8.54%	98.31%	8%	4%	3883562	99.00%	0.84%	0.09%	0.06%
**P7B1**	49518204	85.75%	83.49	35.33%	67.19%	18%	9%	20712958	98.98%	0.85%	0.11%	0.06%
**P7B2**	46121858	83.53%	83.21	34.65%	60.02%	17%	9%	18910438	98.99%	0.84%	0.11%	0.06%
**P7B3**	58598593	85.69%	82.87	34.24%	81.27%	19%	10%	23710326	98.99%	0.84%	0.11%	0.06%
**P7S1**	66036696	84.30%	83.84	38.28%	89.37%	21%	10%	29950121	99.01%	0.83%	0.10%	0.06%
**P7S2**	56877838	84.34%	83.30	37.91%	79.41%	20%	9%	25495488	99.01%	0.83%	0.11%	0.06%
**P7S3**	55570228	85.17%	82.73	38.94%	76.44%	18%	9%	25793768	98.99%	0.84%	0.11%	0.07%

e

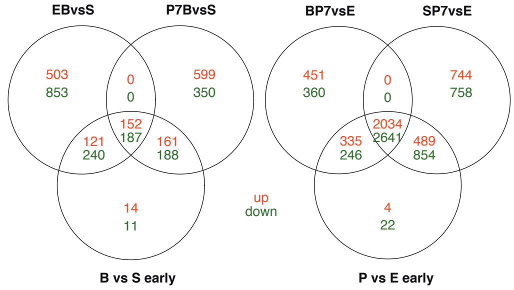

f

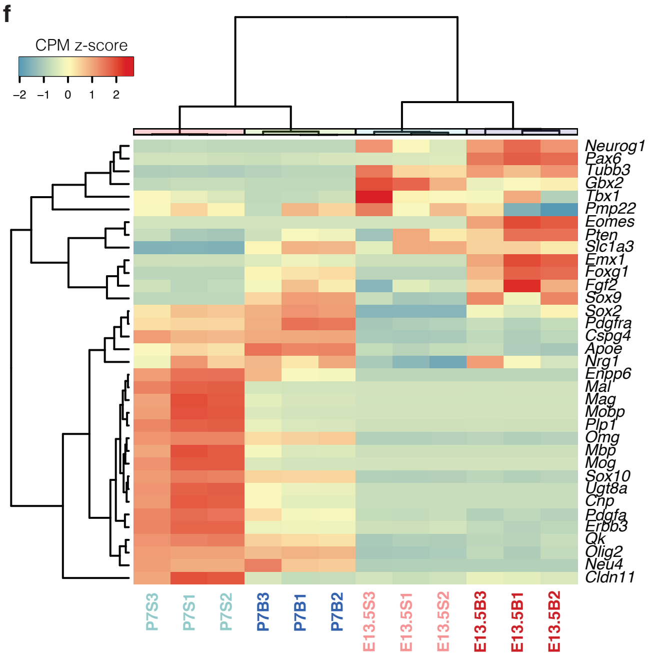

g

	Pre QC	Post QC
Pdgfra+/GFP	2496	1514
Marques et al 2016	526	460

Clusters	# cells
OPC1a	446
OPC1b	264
OPC2	86
COP	193
NFOL	144
Cycling	18
NP1a	82
NP1b	14
NP2	150
NP3	121
eVLMC	214
PLC	24
pnVLMC	148
VLMC	52

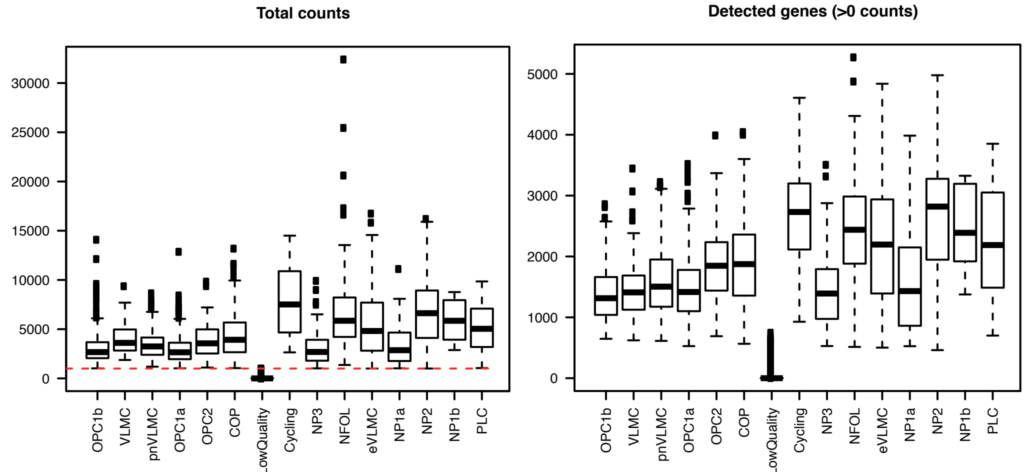

Figure S1 (related to Figures 1-2)

a) FACS sorting graph, highlighting selected Pdgfra+/GFP cells for bulk RNA-Seq;

b) FACS gating of GFP+ cells (FITC) from the P7 spinal cord of Pdgfra-H2B-GFP mice in 2 independent single cell RNA-Seq experiments.

c) FACS gating of GFP+ cells (FITC) from the P7 brain of Pdgfra-H2B-GFP mice in a single cell RNA-Seq experiment.

d) Bulk sequencing library depth and mapping statistics

e) Overlap between differentially expressed genes at each of the stages and regions investigated in the bulk RNA-Seq;

f) Heatmap showing hierarchical clustering of bulk samples based on normalised gene expression (counts per million mapped reads, cpmm) of selected genes involved in oligodendrocyte lineage progression;

g) Single-Cell RNA sequencing Quality Control.

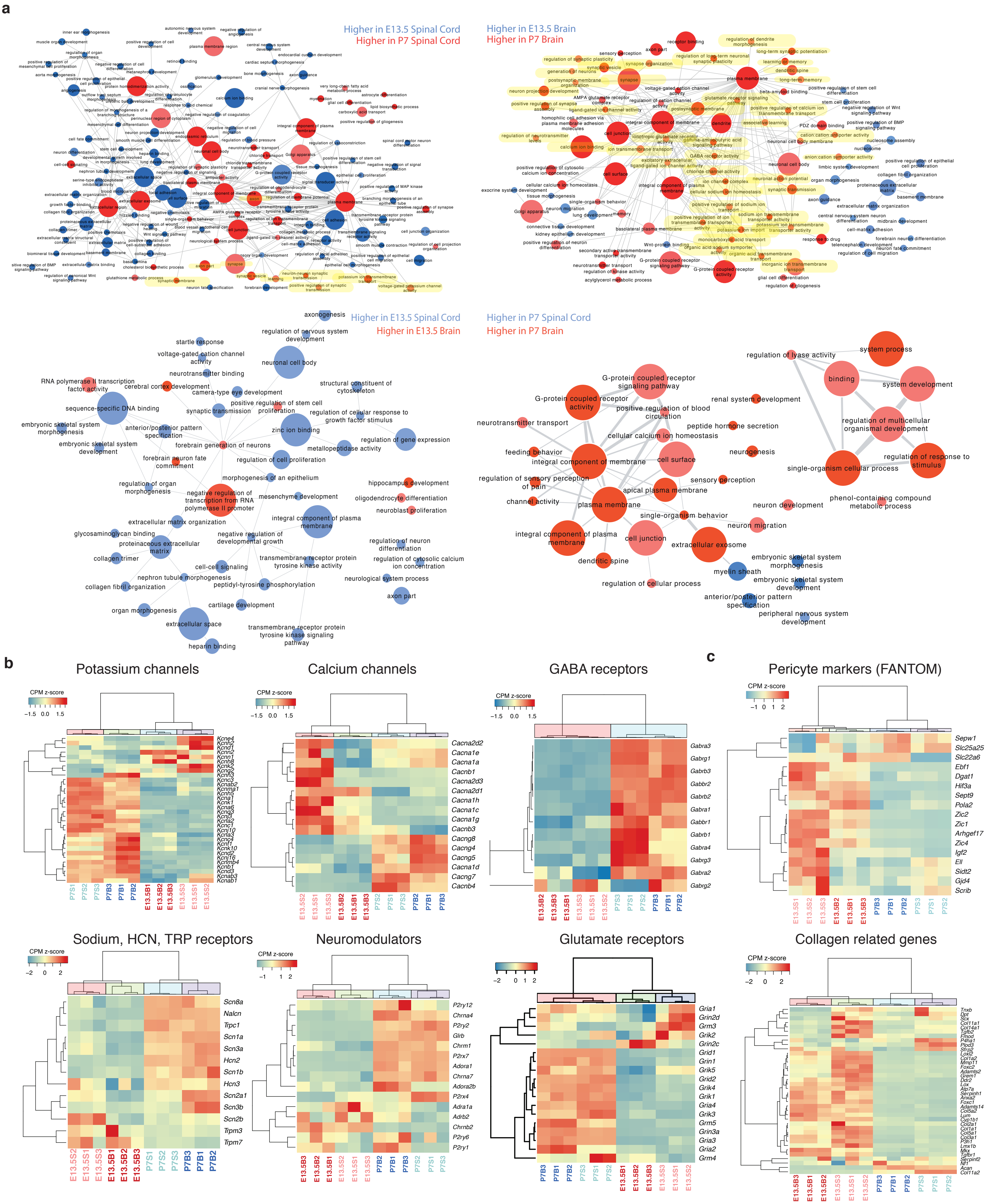

Figure S2 (related to Figure1)

a) Gene ontology analysis with enriched biological functions overrepresented in either E13.5 versus P7 brain, and E13.5 versus P7 spinal cord.

b) Hierarchical clustering of bulk samples based on normalised gene expression (cpmm) of potassium and calcium channels, GABA, Sodium, HCN and TRP receptors and neuromodulator-related, glutamate receptor genes observed in the bulk sequencing in Pdgfra+/GFP cells;

c) Hierarchical clustering of bulk samples based on normalised gene expression (cpmm) of pericyte marker and collagen related genes observed in the bulk sequencing of Pdgfra+/GFP cells

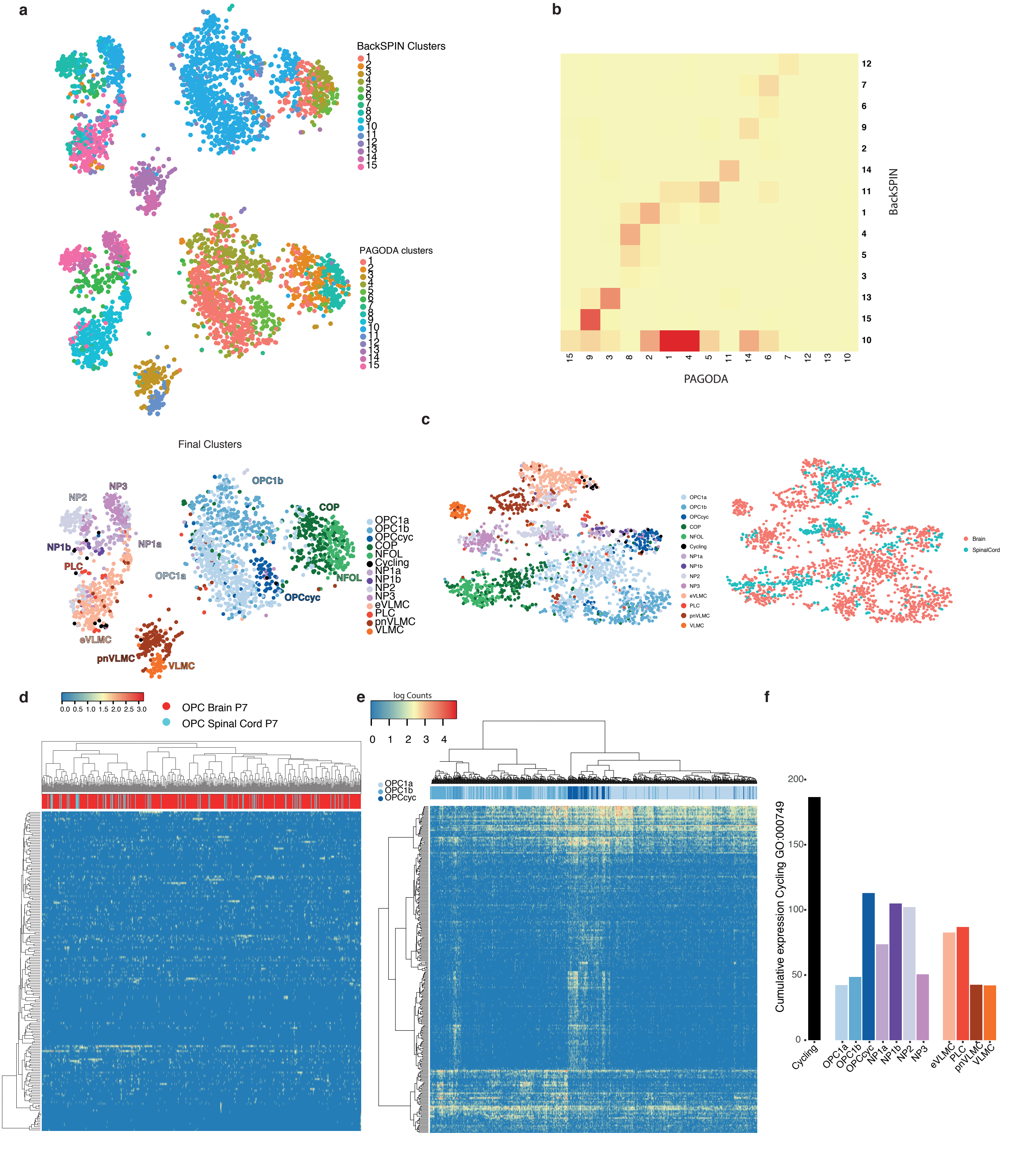

Figure S3 (related with Figure 2)

a) Differences and commonalities in the BackSPIN2 and PAGODA derived clusters, and the merged and final clusters as shown by tSNE;
b) Cluster overlap comparing PAGODA and BackSpin2, colorshaded by number of cells;
c) Noise corrected t-SNE after removal of hidden confounding factors using the f-scLVM package
d) Hierarchically clustered heatmap of top differentially expressed genes between Brain and Spinal Cord P7 OPCs, as assessed by SCDE
e) Hierarchically clustered heatmap of top differentially expressed genes between OPC1a, OPC1b, and OPCcyc, as assessed by SCDE
f) Total molecule count of cell cycle gene expression taken from GO:000749 among the different cell populations;

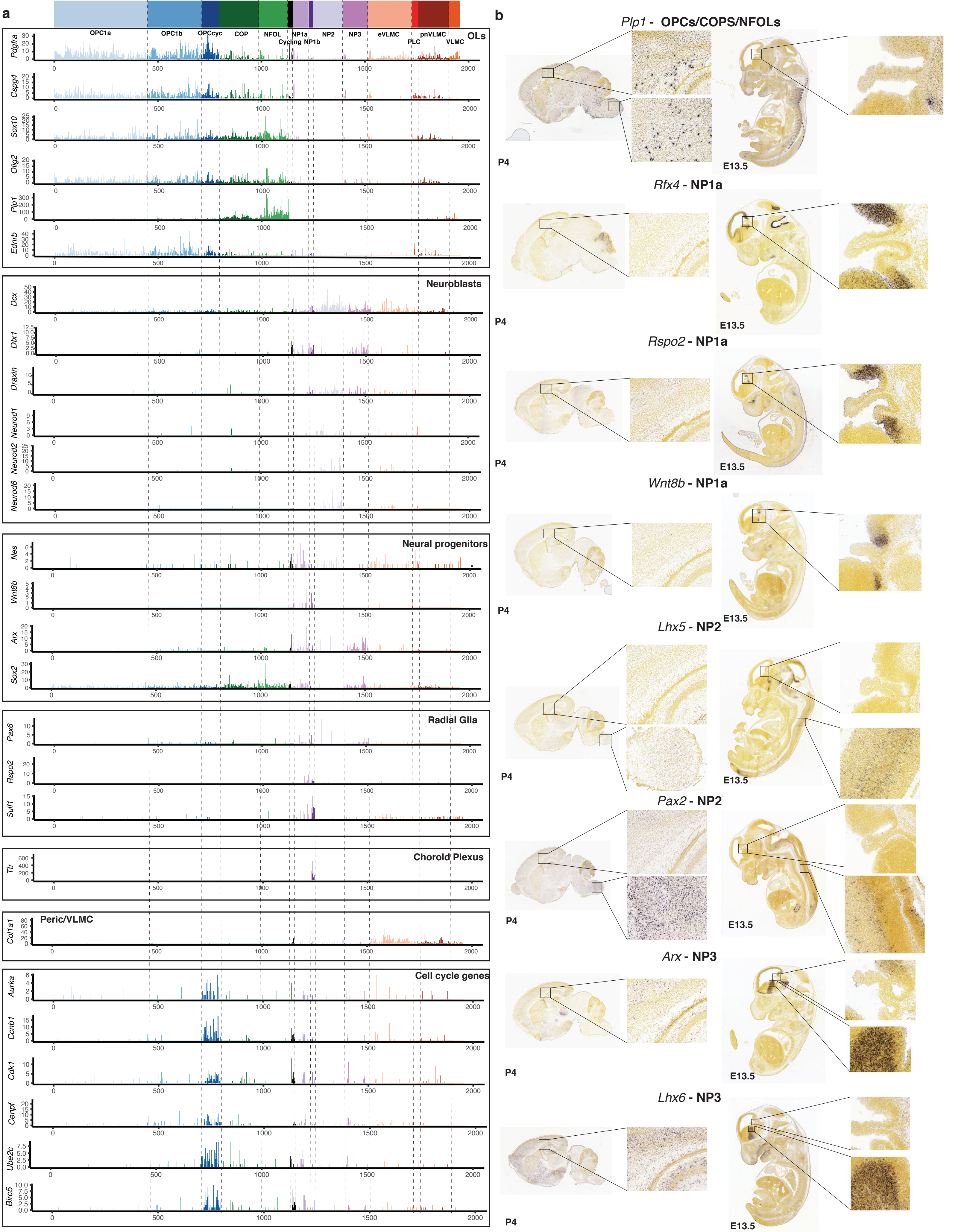

Figure S4 (related to Figure 2) a) Single cell expression of known markers of different lineages in the different clusters, and of cell cycle genes.
b) In situ hybridisation of NP markers at E13.5 and P4 mouse CNS, from @2013 Allen Developing Mouse Brain Atlas

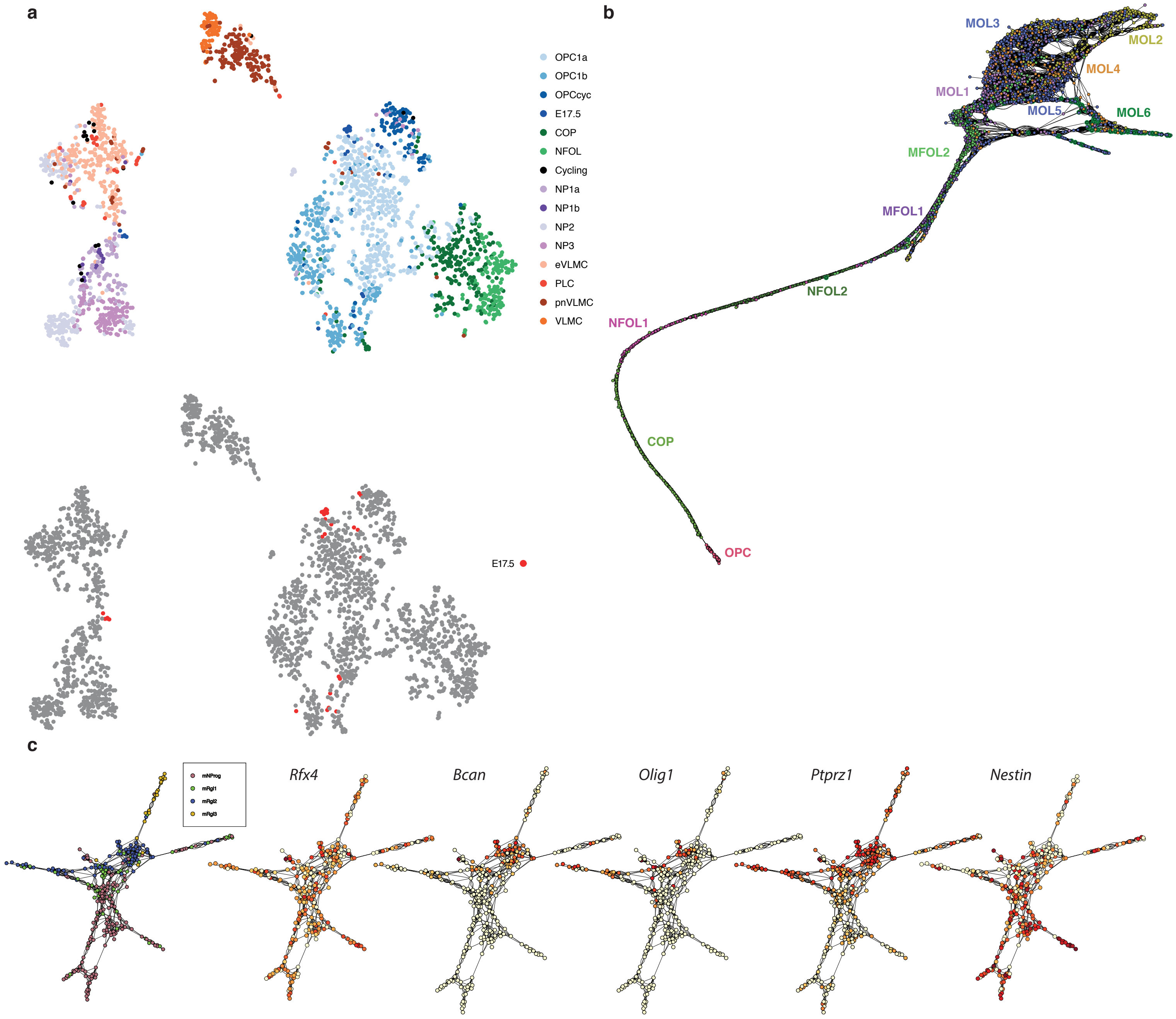

Figure S5 (related to Figure 3)
a) t-SNE representation of single cell data as in Figure 2c, including in addition E17.5 Pdgfra+/GFP cells
b) SCN3E network analysis of the 12 OL lineage populations identified in Marques et al 2016, as a proof of concept of the SCN3E; Myelin forming oligodendrocytes (MFOL)1 was suggested to be upstream mature oligodendrocytes MOL1, while MFOL2 was connected to MOL5;
c) SCN3E network analysis of embryonic ventral midbrain single-cell RNA-Seq from La Manno et all, 2016, with a focus on *Rfx4*+ populations

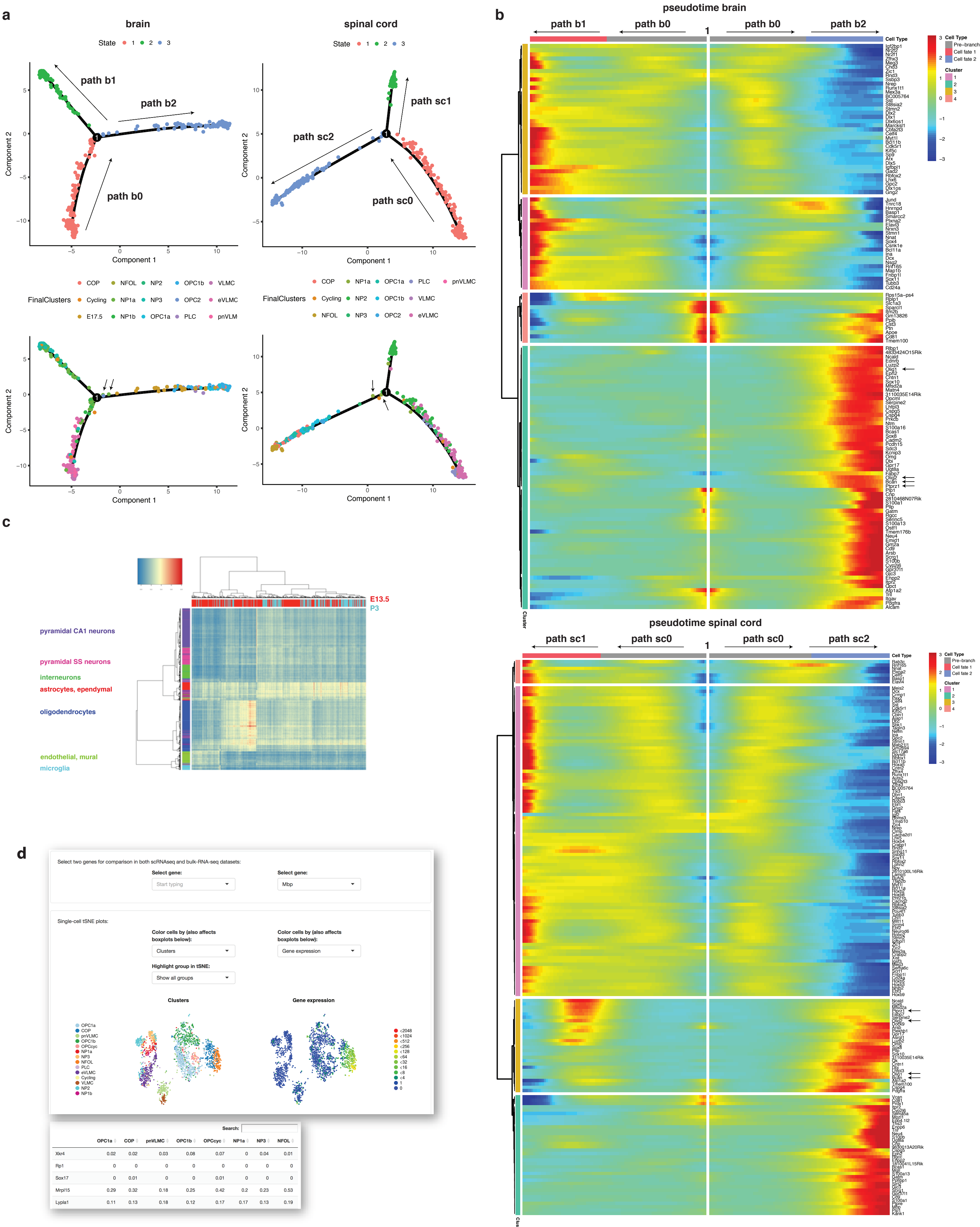

Figure S6 (related to Figures 3 and 4)

a) Reverse graph embedding deconvolved path depicting the captured differentiation trajectories in the single cell data from brain (left) and spinal cord (right) by Monocle2. Paths in the brain (b0/1/2) and spinal cord (s0/1/2) are shown as inferred by monocle, where the numbers 0, 1, and 2 stand for VLMC-, Neuronal-, and Oligodendrocyte- fates respectively. b) Differential expression heatmap capturing the most differentially expressed genes related to the oligodendrocyte (path 2) and neuronal lineage (path 1), with respect to the inferred branches in Monocle2. c) Pearson correlation performed on P7/8 single-cell RNAseq dataset obtained from a lineage tracing experiment with Pdgfra-Cre-ERT/RCE mice upon injection of tamoxifen at E12.5/13.5 and P3/P4 and Zeisel et al., 2015 single-cell RNA-Seq dataset. d) Screenshots of the resource website app, illustrating the exploration of expression data and table browsing.
