# Supplementary material for: Transcriptional Convergence of Oligodendrocyte Lineage Progenitors during Development
Source: Dev Cell. 2018 Aug 20;46(4):504–517.e7. doi: 10.1016/j.devcel.2018.07.005 (PMC6104814; doi:10.1016/j.devcel.2018.07.005)
Supplement: Document S2. Article plus Supplemental Information [file mmc6.pdf]

Resource

Developmental Cell

Transcriptional Convergence of Oligodendrocyte Lineage Progenitors during Development

Graphical Abstract

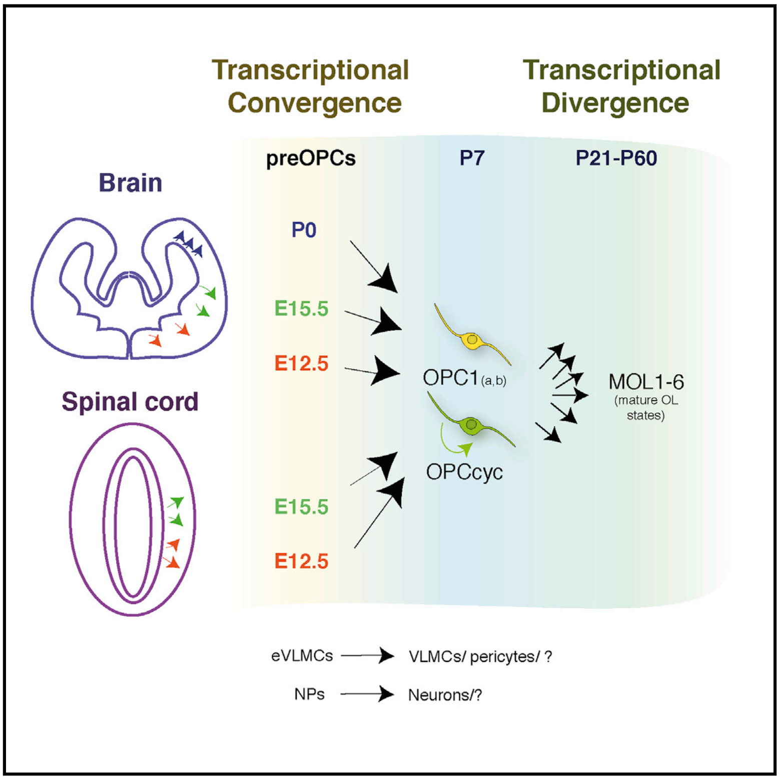

Authors

Sueli Marques, David van Bruggen, Darya Pavlovna Vanichkina, ..., Jens Hjerling-Leffler, Ryan James Taft, Gonçalo Castelo-Branco

Correspondence

goncalo.castelo-branco@ki.se

In Brief

Marques, van Bruggen, Vanichkina et al. present a bulk and single-cell transcriptomics resource of mouse forebrain and spinal cord *Pdgfra+* cells at embryonic and post-natal stages. Embryonic *Pdgfra+* cells produce distinct post-natal cell lineages. Post-natal oligodendrocyte precursor cells arising from different CNS regions are ultimately highly similar.

Highlights

- OPCs arising from different parts of the CNS are highly similar to one another
- Pre-OPCs converge into similar transcriptional and electrophysiological OPC states
- E13.5 *Pdgfra+* cells give rise mainly to OPCs in the post-natal CNS
- E13.5 *Pdgfra+* cells also give rise to cells of the pericyte lineage

Data Resources

GSE95194
GSE95093

Marques et al., 2018, Developmental Cell *46*, 504–517
August 20, 2018 © 2018 The Author(s). Published by Elsevier Inc.
https://doi.org/10.1016/j.devcel.2018.07.005

Developmental Cell
Resource

Transcriptional Convergence of Oligodendrocyte Lineage Progenitors during Development

Sueli Marques,[1,8] David van Bruggen,[1,8] Darya Pavlovna Vanichkina,[2,3,8] Elisa Mariagrazia Floriddia,[1] Hermany Munguba,[1] Leif Väremo,[5] Stefania Giacomello,[6] Ana Mendanha Falcão,[1] Mandy Meijer,[1] Åsa Kristina Björklund,[7] Jens Hjerling-Leffler,[1] Ryan James Taft,[3,4] and Gonçalo Castelo-Branco[1,9,*]
[1]Laboratory of Molecular Neurobiology, Department Medical Biochemistry and Biophysics, Biomedicum, Karolinska Institutet, Stockholm 17177, Sweden
[2]Gene and Stem Cell Therapy Program, Centenary Institute, University of Sydney, Camperdown, NSW 2050, Australia
[3]Institute for Molecular Bioscience, University of Queensland, St Lucia, QLD 4067, Australia
[4]Illumina, Inc., San Diego, CA 92122, USA
[5]Science for Life Laboratory, Department of Biology and Biological Engineering, Chalmers University of Technology, Kemivägen 10, Göteborg 412 96, Sweden
[6]Science for Life Laboratory, Department of Biochemistry and Biophysics, Stockholm University, Box 1031, 17121 Solna, Sweden
[7]Science for Life Laboratory, Department of Cell and Molecular Biology, Uppsala University, Husargatan 3, 75237 Uppsala, Sweden
[8]These authors contributed equally
[9]Lead Contact
*Correspondence: goncalo.castelo-branco@ki.se
https://doi.org/10.1016/j.devcel.2018.07.005

SUMMARY

Pdgfra+ oligodendrocyte precursor cells (OPCs) arise in distinct specification waves during embryogenesis in the central nervous system (CNS). It is unclear whether there is a correlation between these waves and different oligodendrocyte (OL) states at adult stages. Here, we present bulk and single-cell transcriptomics resources providing insights on how transitions between these states occur. We found that post-natal OPCs from brain and spinal cord present similar transcriptional signatures. Moreover, post-natal OPC progeny of E13.5 *Pdgfra*+ cells present electrophysiological and transcriptional profiles similar to OPCs derived from subsequent specification waves, indicating that *Pdgfra*+ pre-OPCs rewire their transcriptional network during development. Single-cell RNA-seq and lineage tracing indicates that a subset of E13.5 *Pdgfra*+ cells originates cells of the pericyte lineage. Thus, our results indicate that embryonic *Pdgfra*+ cells in the CNS give rise to distinct post-natal cell lineages, including OPCs with convergent transcriptional profiles in different CNS regions.

INTRODUCTION

Oligodendrocytes (OLs; for abbreviations, please refer to Table S4) are one of the most abundant cell types in the central nervous system (CNS). OLs have been classically described as support cells for neurons, responsible for the insulation of axons and enabling rapid saltatory conduction, although recent findings suggest their involvement in other processes (Larson et al., 2018; Micu et al., 2016; Nave and Werner, 2014). OLs arise from the differentiation of oligodendrocyte precursor cells (OPCs). Since OLs and OPCs are found throughout the CNS, they were originally thought to be derived from embryonic neural progenitors (NPs) from nearby ventricular zones or from radial glial cells in all CNS regions (Hirano and Goldman, 1988). However, *in vitro* experiments with cell suspensions and explants suggested a restricted ventral origin of OPCs in the embryonic rat spinal cord (Warf et al., 1991). OPCs expressing platelet-derived growth factor receptor alpha (*Pdgfra*) appeared to arise exclusively from ventral domains of the CNS, with a subset migrating to dorsal regions (Pringle and Richardson, 1993). This led to the hypothesis of a single embryonic lineage for OPCs, arising at embryonic day (E) 12.5 from progenitor domains and dependent on sonic hedgehog (Shh) (Richardson et al., 2000). Nevertheless, cells expressing *Plp/dm-20* were also observed to give rise to OLs (Spassky et al., 2000), suggesting that other progenitors in the dorsal/ventral axis of the CNS could be alternative sources of OPCs. Indeed, knockdown of transcription factors (TFs) involved in the specification of ventral spinal cord domains and lineage-tracing studies uncovered a subset of OPCs originating from the mouse dorsal spinal cord at E14.5-E15.5 (Cai et al., 2005; Fogarty et al., 2005; Vallstedt et al., 2005). Dorsal-derived spinal cord OPCs were not dependent on Shh for their specification (Cai et al., 2005; Vallstedt et al., 2005). Moreover, they populated specific regions of the spinal cord, while OPCs from the ventral-derived neuroepithelium gave rise to the vast majority of OLs throughout the adult spinal cord (Tripathi et al., 2011; Fogarty et al., 2005). A similar pattern of OL specification was found to operate in the mouse forebrain. The first OPCs were shown to be formed at E12.5 from *Nkx2.1*-expressing precursors in the medial ganglionic eminence, followed by a subsequent wave from precursors expressing *Gsx2* in the lateral and medial ganglionic eminences at E15.5 (Kessaris et al., 2006). The last wave of cortically derived *Emx1*-expressing precursors begins

 © 2018 The Author(s). Published by Elsevier Inc.
This is an open access article under the CC BY license (http://creativecommons.org/licenses/by/4.0/).

developing at birth and eventually becomes the major contributor to the post-natal OPC pool (Kessaris et al., 2006).

It is unclear whether diverse populations and developmental waves of OPC in the CNS generate identical or distinct OLs. When Richardson and colleagues specifically ablated OPCs generated from three brain developmental waves in mice, no gross behavior abnormalities were observed, most likely due to functional compensation of the lost populations by OL lineage cells from non-ablated regions (Kessaris et al., 2006). This suggested that OL lineage cells derived from different brain regions are, or can become, functionally equivalent. However, adult OPCs have been classified into different sub-populations with diverse cycling (Jang et al., 2013; Young et al., 2013; Clarke et al., 2012), myelinating (Viganò et al., 2013), and remyelination (Crawford et al., 2016) properties. In addition, spinal cord post-natal OPCs give rise to OLs that present a higher myelin sheet length than OLs from the cortex (Bechler et al., 2015). By performing single-cell RNA-seq (RNA sequencing) on ~5,000 cells of the OL lineage in juvenile and adult mice, we found at least six distinct mature OL cell states (Marques et al., 2016), suggesting possible functional heterogeneity.

Diverse adult cortical interneuron populations have recently been shown to be specified already at the embryonic stages in the CNS (Mayer et al., 2018; Mi et al., 2018). In order to investigate whether different embryonic waves of OPC specification give rise to redundant OLs or are assigned to specific mature OL states and/or populations, we analyzed the transcriptome of mouse forebrain and spinal cord *Pdgfra*+ cells at E13.5, E17.5, P7, and juvenile/adult stages using bulk and single-cell RNA-seq (resource website with browsable graphic visualizations of the data and annotated tables (Figure S6D) available at https://ki.se/en/mbb/oligointernode. Features as expression profile of genes of interest in the different clusters, tissues and ages can be explored. Gene expression tables can also be explored to sort for genes differentially expressed in different clusters). E13.5 *Pdgfra*/GFP+ cells were found to constitute six distinct cell states within the OL, pericyte, and possibly other lineages. Embryonic *Pdgfra*/GFP+ cells expressed patterning TFs that were diagnostic of their sites of origin in the ventricular zone (VZ) and other transcripts typical of NPs. These cells might be transitional cells on the path to becoming OPCs (“pre-OPCs”), or, alternatively, they might be multi-potent or bi-potent NPs that still retain the ability to generate neurons and glia. Strikingly, expression of patterning TFs was greatly attenuated at post-natal ages, when a common transcriptional profile emerged that was characteristic of post-natal OPCs and compatible with electrophysiological capacity. Thus, the transcriptional program of post-natal OPCs is independent of when and where in the embryonic VZ they were originally specified (e.g., spinal cord versus brain, dorsal versus ventral VZ).

In addition to OPCs, we detected a group of *Pdgfra/GFP*+ cells in the embryonic and post-natal spinal cord and brain that had the transcriptional character of pericytes. These cells might have been generated from the *Pdgfra/GFP*+ NPs described above or from a separate source, for example, from cells associated with blood vessels that invade the developing brain and spinal cord from the pial surface before birth.

In sum, our results indicate that while embryonic *Pdgfra/GFP*+ cells in the CNS are heterogeneous, spatial and temporal transcriptional convergence occurs in the transition between embryonic pre-OPCs and OPCs during development, with OPCs arising in different parts of the embryonic germinal zones being ultimately highly similar to one another.

RESULTS

Transcriptional Profiles of *Pdgfra/GFP*+ Cells during CNS Development

To determine the transcriptional profile of progenitors of the OL lineage during development, we performed stranded total RNA-seq of cells expressing *Pdgfra*, a widely used marker for OPCs in CNS, isolated by Fluorescence-Activated Cell Sorting (FACS) GFP+ populations from the (fore) brain or spinal cord of *Pdgfra*-H2B-GFP mice (Klinghoffer et al., 2002) at E13.5 and P7 (Figures 1A and S1A–S1E). Differential gene expression analysis revealed increased levels of differentiation and/or myelination-related genes (*Mbp*, *Plp1*, *Mag,* and *Mog*, among others) in *Pdgfra*/GFP+ cells at P7 relative to E13.5, and in the spinal cord when compared to the brain (*Opalin* and *Plp1*, Figure 1B; Table S1). P7 spinal cord *Pdgfra*/GFP+ cells were characterized by higher expression of genes corresponding to later stage differentiation (*Mog, Mal, Mag*) when compared to P7 brain, which in contrast exhibit increased expression of *Pdgfra, Sox2, and Sox9* (Figure S1F and Table S1). This pattern of expression would suggest that OPCs are more prone to differentiation in the spinal cord, consistent with myelination occurring earlier in the spinal cord compared to brain (Marques et al., 2016; Coffey and McDermott, 1997).

Many TFs involved in patterning and cell specification (*Hox and Lhx* genes, *Otx2, Nkx2.1, Arx*) were expressed in *Pdgfra*/GFP+ cells at E13.5 and substantially down-regulated or absent at P7 (Figure 1C and Table S1). In contrast, TFs with roles in OL differentiation (*Sox10*, *Sox8*, *Nkx2.2*) were enriched at P7, when compared to E13.5. Principal-component analysis confirmed that *Pdgfra*/GFP+ populations were more distinct temporally than spatially (Figure 1D). Gene ontology (GO) analysis indicated that, apart from genes involved in differentiation and/or myelination, spinal cord cells were enriched in genes with TF activity (Figure 1E–highlighted in blue, Figure S2A and Table S1), consistent with the expression of patterning TFs involved in anterior/posterior regionalization (Figure 1B). TF activity was also enriched in E13.5 *Pdgfra*+/GFP cells compared to P7 (Figure 1F). Post-natal *Pdgfra*/GFP+ cells, in particular in the forebrain, were enriched in genes involved in ion channel complex and transport, and synaptic transmission, among others (Figures 1F and S2A–highlighted in yellow). Some of the contributions to these P7 signatures were potassium and sodium ion channels, as well as glutamate receptor subunits and (gamma-Aminobutyric acid) GABA receptors (Figure S2B). In contrast to post-natal cells, E13.5 *Pdgfra*/GFP+ cells were enriched in genes involved in a myriad of unrelated processes, such as neural precursor and neuron development and/or specification, extracellular matrix and collagen organization, and basal lamina and angiogenesis (Figure 1F–highlighted in green and Figures S2A and 2C). This diversity of biological function was intriguing, since these cells are thought to give rise exclusively to cells of the OL lineage.

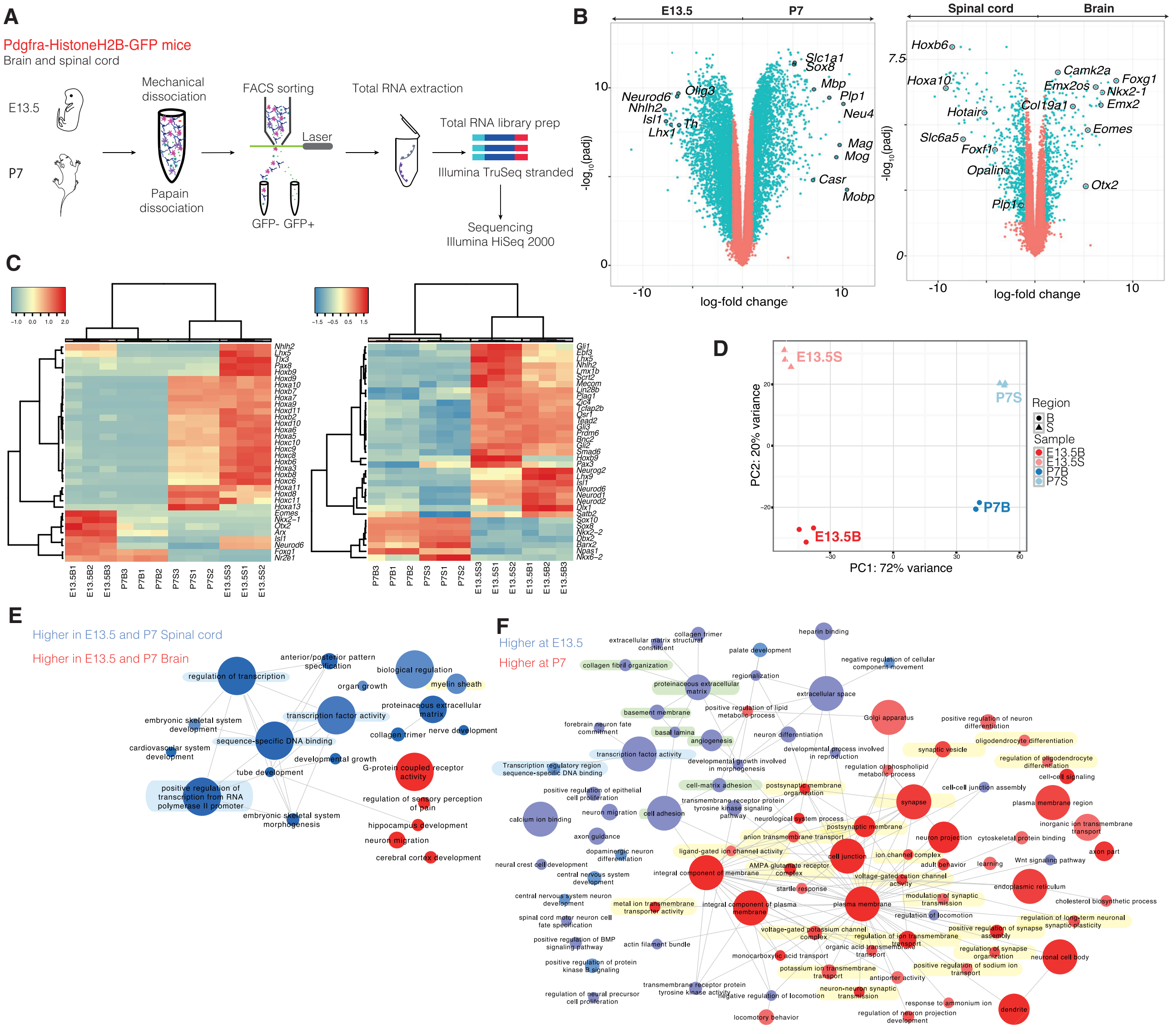

Figure 1. Temporal and Spatial Transcriptional Heterogeneity of *Pdgfra*/GFP+ Cells

(A) Schematic of *Pdgfra*/GFP+ cell purification for bulk RNA sequencing.

(B) Volcano plots of Gencode-annotated genes depicting differential expression between (fore) brain versus spinal cord, and E13.5 versus P7.

(C) Hierarchical clustering of bulk samples based on normalized gene expression (cpmm) of transcription factors annotated in animalTFDB.

(D) Principal-component analysis of bulk RNA-seq of *Pdgfra*/GFP+ cells from E13.5 and P7 (fore) brain and spinal cord.

(E and F) Gene ontology analysis of enriched biological functions overrepresented in either (fore) brain versus spinal cord (E), or E13.5 versus P7 (F).

See also Figures S1, S2 and Tables S1.

Single-Cell RNA-Seq Reveals Similar Transcriptional Profiles of Post-natal OPCs in the Spinal Cord and Brain

Since the diversity of biological processes associated with E13.5 *Pdgfra*+/GFP cells could reflect a broad cell potential or cell heterogeneity, and the latter would be obscured by bulk RNA-seq analysis, we performed single-cell RNA-seq using STRT-Seq technology (Islam et al., 2014) on 2496 *Pdgfra*+/GFP cells (1,514 cells after quality control [QC]) from *Pdgfra*-H2B-GFP (Klinghoffer et al., 2002) and *Pdgfra*-CreERT-RCE (LoxP-GFP) mice (Kang et al., 2010) at E13.5 and P7 (Figures 2A and S1G). For comparison purposes, we also included 271 OPCs, 114 committed oligodendrocyte precursors (COPs), and 75 vascular and leptomeningeal cells (VLMCs) from the juvenile and adult CNS (Marques et al., 2016) in the analysis.

There was a clear temporal segregation of E13.5 and P7 cells (Figure 2B) in accordance with the bulk RNA-seq data (Figure 1D). Spatial segregation was also observed, but to a lesser extent than in the bulk analysis. In fact, subsets of P7 brain and spinal cord cells intermingled in the t-Distributed Stochastic Neighbor Embedding (t-SNE plot), suggesting close similarity (Figure 2B). We performed cell clustering using two different algorithms, BackSPIN2 (Marques et al., 2016) and PAGODA (Fan

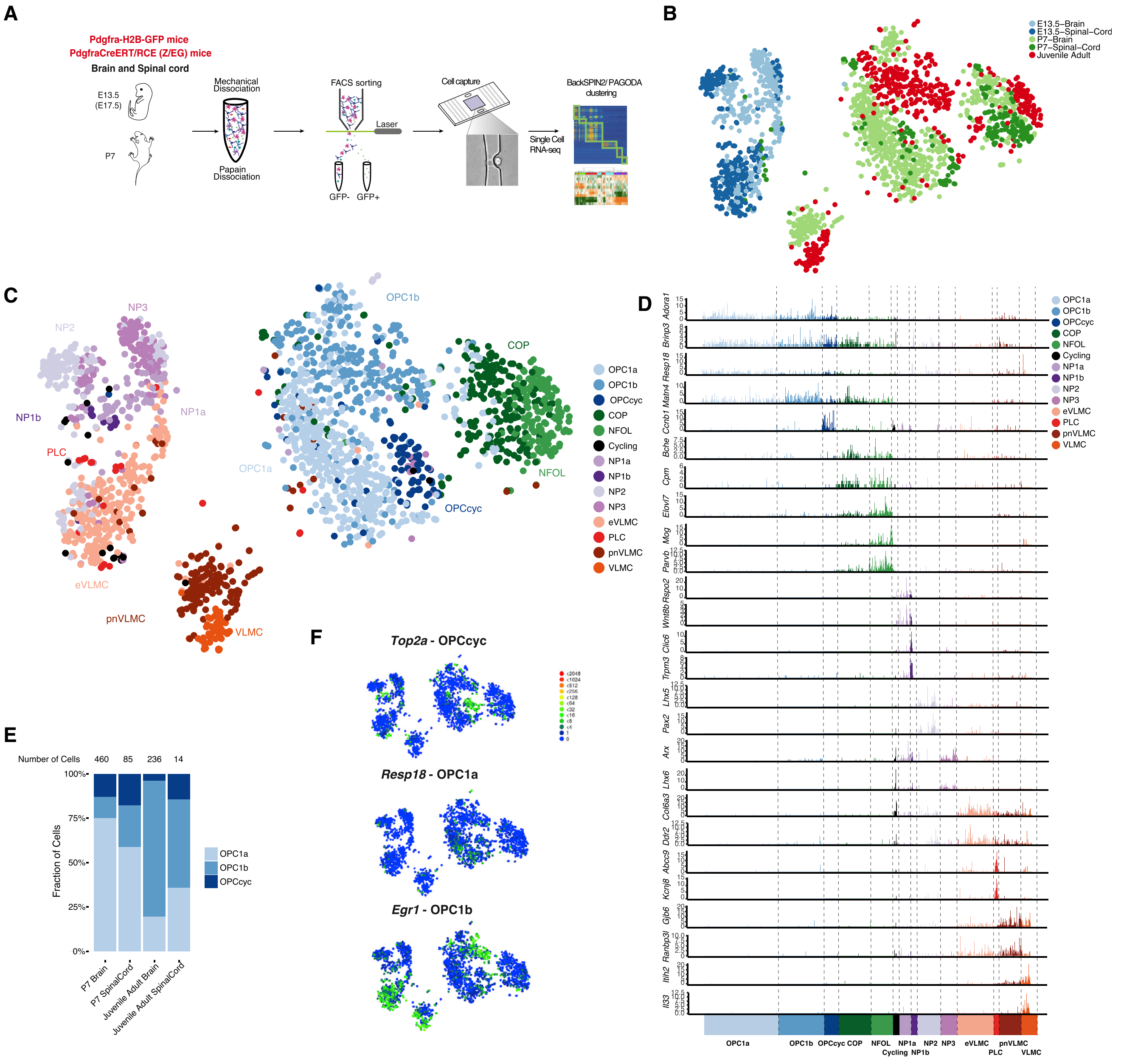

Figure 2. Single-Cell RNA-Seq Reveals Similar Transcriptional Profiles of OPCs in the Post-natal Spinal Cord and Brain

(A) Scheme of Pdgfra/GFP+ cell purification for single-cell RNA-seq.

(B and C) t-SNE of single-cell RNA-seq of E13.5, P7 *Pdgfra*/GFP+ cells, and P20–30, and P60 cells from Marques et al., highlighting age and region (B) and identified clusters (C).

(D) Single-cell expression of the most relevant marker genes in all identified populations.

(E) Fraction of OPC1a, 1b and OPCcyc in each P7, juvenile/adult tissues.

(F) Single-cell expression of cluster-specific genes for OPCs, overlayed in t-SNE from Figure 2C.

See also Figures S1, S3, S4 and Tables S2 and S3.

et al., 2016). These algorithms revealed similar clusters (Figures S3A and S3B), which were merged to form our final cluster set (see STAR Methods for further details). We could identify cells expressing hallmarks of COPs (*Bmp4, Neu4*) and newly formed OLs (NFOL) (*Prom1, Tspan2*) from the P7 CNS (Figures 2B and 2C), confirming that these populations appear to arise in the CNS already at P7 and remain transcriptionally similar in the juvenile and adult CNS (Marques et al., 2016). COPs, and particularly NFOLs, were found in higher proportions in the spinal cord at P7, highlighting that the process of differentiation is further advanced in this region (Figures 2B and 2C).

Three clusters of P7 and juvenile/adult cells presented markers of OPCs (OPC1a, 1b, and OPCcyc), (Figures 2C, S4A, and Table S2). Surprisingly, P7 OPCs from spinal cord and brain

clustered together (Figures 2B and 2C). In order to rule out that this similarity is not due to hidden confounding factors, including noise and putative batch effects, we performed a removal of confounding factors using the f-scLVM package (Buettner et al., 2017), which revealed an even more homogeneous distribution of spinal cord- and brain-derived OPCs (Figure S3C). Furthermore, batch-corrected Bayesian based single cell differential expression (SCDE) analysis (Fan et al., 2016) of the single-cell OPC populations in the P7 brain and spinal cord failed to identify significant differences in gene expression (Figure S3D). To more conclusively exclude major differences between brain and spinal cord OPCs, we used Model-based Analysis of Single Cell Transcriptomics (MAST) (Finak et al., 2015), a differential expression algorithm that, in contrast to the Bayesian-based SCDE, uses a hurdle model to calculate significant differential expression. MAST analysis yielded a limited number of genes significantly differentially expressed after multiple testing corrections (Table S3). With the exception of *Hoxc8*, patterning genes present in E13.5 *Pdgfra*/GFP+ cells were not significantly differentially expressed or sporadically expressed (Table S3). Likewise, with the exception of *Plp1*, myelination genes enriched in P7 spinal cord *Pdgfra*/GFP+ cells in the bulk RNA-seq, such as *Klk6*, *Trf*, *Mbp*, *Cnp*, *Qk*, and *Opalin*, were not differentially expressed in P7 spinal cord and brain OPCs (Table S3).

Our single-cell RNA-seq analysis indicates that P7 OPCs from different CNS regions, such as the brain and spinal cord, present comparable gene expression profiles, in contrast to the bulk RNA-seq analysis of the same populations (Figure 1). The difference in differentiation and/or myelination genes in the bulk populations (Figure 1B) is unlikely to reflect intrinsic differences in myelinating potential between OPCs, and might rather reflect that the half-life of H2B-GFP protein is longer than PDGFRA mRNA, resulting in the GFP labeling of other populations within the OL lineage. Indeed, COPs, and particularly NFOLs, are present in higher numbers in the spinal cord *Pdgfra*/GFP+ FACS-sorted populations when compared to brain *Pdgfra*/GFP+ populations (Figures 2B and 2C), which would explain the enrichment of differentiation and/or myelination genes in spinal cord populations in the bulk RNA-seq. Thus, single-cell RNA-seq analysis allows the deconvolution of our bulk RNA-seq data and suggests a loss of patterning factors and subsequent transcriptional convergence of OPC cell states in the different anterior-posterior regions at P7.

The three OPC clusters presented unique temporal distributions, with none being present in the E13.5 (Figures 2B and 2C), OPC1a enriched at P7, and OPC1b at juvenile/adult CNS (Figure 2E). OPCcyc was segregated from OPC1a/b and expressed genes involved in mitosis and cell division (Figure S3F). OPC1a and OPC1b clusters were quite similar in their transcriptional profile (Figures S3E and S4A), which could indicate that they might constitute cell states rather than cell types or even constitute a single cluster. However, OPC1a presented distinctive expression of *Resp18* (Figures 2D and 2F) and genes involved mainly in diverse metabolic processes (mitochondrial energy production, RNA processing), such as ATP synthase, *Cox,* and ribosomal genes (Table S2). OPC1b was characterized by the expression of genes involved in nervous system development and transcription regulation. We also found enriched expression of immediate early genes such as *Fosb*, *Fos*, *Jun*, and *Egr1* (Figure 2F; Table S2), which might be associated with specific activation of OPC1b cells by the neighboring neuronal network (Hrvatin et al., 2018), although it could also simply reflect cellular stress during the cell extraction procedure (van den Brink et al., 2017). All three OPC cell states were observed in both brain and spinal cord (Figure 2E), highlighting the similarity of OPCs in the anterior-posterior axis of the CNS.

E13.5 *Pdgfra+*/GFP Cells Do Not Have the Hallmarks of Post-natal OPCs

Clustering and differential gene expression analysis allowed the identification of six distinct populations of E13.5 *Pdgfra*/GFP+ cells (Figure 2C; Table S2). Four populations (NPs, 1a, 1b, 2, and 3) presented high correlation between themselves (Figure 3A). We examined the expression profiles of identified markers of these populations in the E13.5 and P4 CNS through publicly available *in situ* hybridization data from the Allen Institute for Brain Science (©2008 Allen Institute for Brain Science. Allen Developing Mouse Brain Atlas available from http://developingmouse.brain-map.org). The NP markers presented non-overlapping expression patterns at E13.5 (Figure S4B), indicating that they are indeed expressed in distinct cell populations. Moreover, we observed absence or reduced expression of some of these markers in the corpus callosum at P4, suggesting that their expression is attenuated or repressed after birth, in agreement with our single-cell data (Figure S4B).

NP1-3 expressed markers of the NP, neuroblast, or radial glia lineages (Figure S4A). To confirm these identities, we performed Pearson correlation comparisons on the embryonic midbrain cell populations identified by single-cell RNA-seq (La Manno et al., 2016). Although correlation was low, this indicated a partial similarity between NPs and neuroblast and neuronal progenitor populations (Figure 3B). While some cells within the NP populations expressed individual markers of OPCs, RNA levels for these genes were relatively low in the cells where they were observed (Figure S4A).

Two additional E13.5 clusters expressed higher levels of *Pdgfra* and *Csgp4* (NG2), albeit lower than P7 OPCs (Figure S4A). They also expressed genes involved in collagen formation, such as *Col1a1*. Correlation analysis with previous published single-cell datasets (La Manno et al., 2016; Marques et al., 2016) indicates that these cells are related to pericytes and VLMCs (Figures 3B and 3C), and as such, they were named embryonic VLMCs (eVLMCs) and pericyte lineage cells (PLCs). Thus, the pericyte/VLMC signature of E13.5 *Pdgfra+*/GFP cells found in the bulk RNA-seq (Figures 1F and S2C) is most likely due to cell heterogeneity rather than broader cell potential.

NP1a Constitutes a Pre-OPC Neural Progenitor Population

NP1a and NP3 were more similar to OPCs than other NPs (Figures 3A and 3C), and as such might be the embryonic progenitors of the OL lineage. Nevertheless, our data indicate that there is a transcriptional leap between E13.5 progenitors and P7 OPCs (Figure 2C). In order to better define the transitions between embryonic and post-natal states, we performed additional single-cell RNA-seq with E17.5 *Pdgfra*/GFP+ cells of Pdgfra-H2BGFP knockin mice. We found that, at this stage, *Pdgfra*/GFP+ cells were mainly associated with OPC clusters,

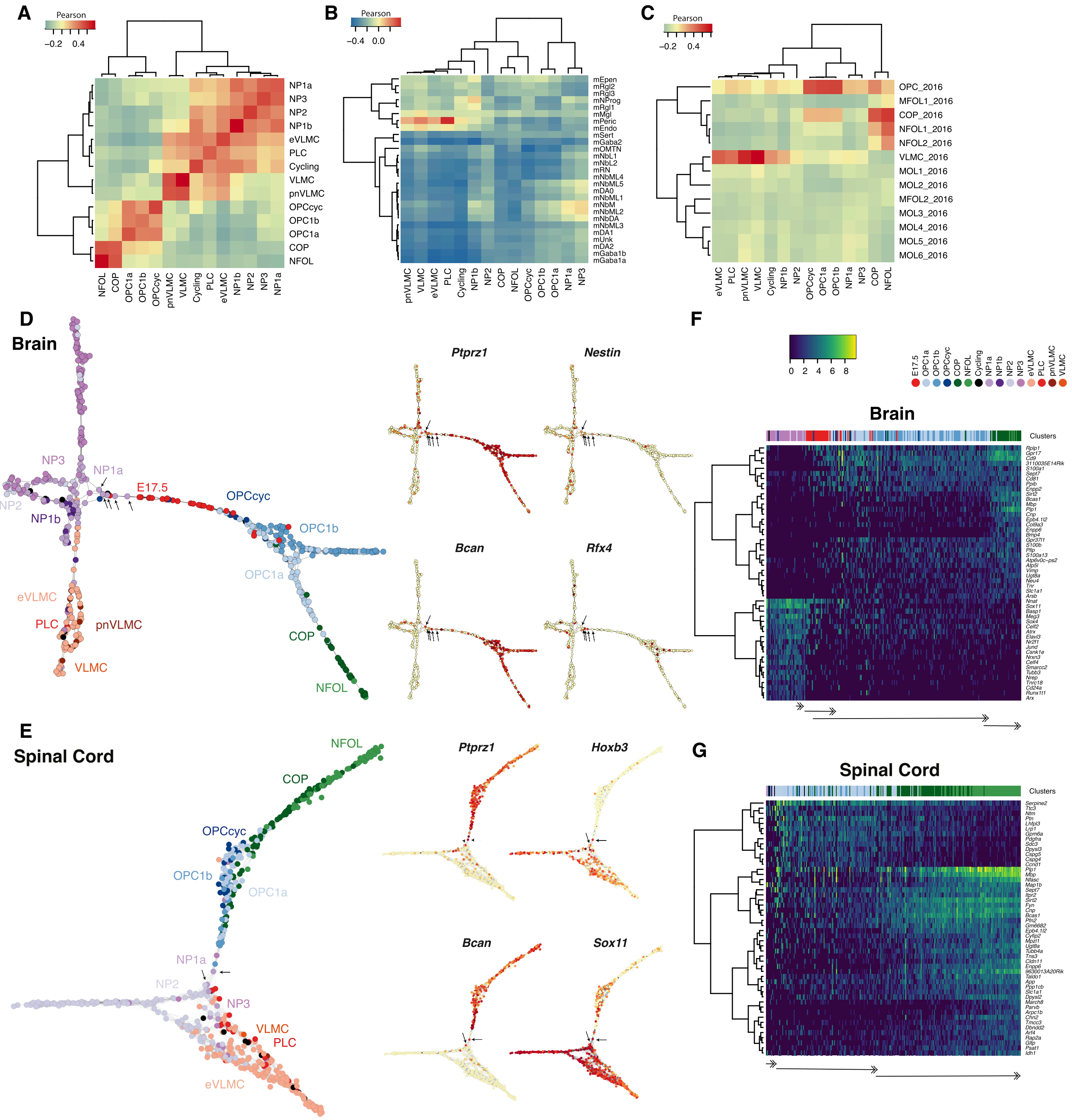

Figure 3. NP1a Constitutes a Pre-OPC Neural Progenitor Population

(A–C) Heatmaps with correlation analysis between all identified populations (A), of all the populations compared to La Manno et al. (2016) dataset (B) and of all the populations compared to Marques et al. (2016) dataset (C).

(D and E) SCN3E network analysis of (fore) brain (D) and spinal cord (E) E13.5 and P7 *Pdgfra*/GFP+ cells, with juvenile/adult OPCs, COPs, and VLMCs from Marques et al. (2016) Brain SCN3E also includes cells from E17.5 scRNA-seq experiment. On the right, overlay of gene expression levels of a subset of genes on the SCN3E graphs, with gradient from yellow (low expression) to red (high expression).

(F and G) Heatmaps of pseudo-time iterations from the SCN3E analysis of brain (F) and spinal cord (G), representing the 50 most variable genes along pseudo-time with at least $p < 0.01$ computed using MAST. Arrows illustrate transitions between pre-OPCs (NP1a), OPCs, and COPs/NFOLs. An additional transition is observed in (fore) brain, corresponding to E17.5 to P7 OPCs.

See also Figures S5 and S6.

although a subset of cells clustered close to E13.5 NP1a cells (Figure S5A). As such, at this stage, our data suggest that NPs are nearly absent within the *Pdgfra*+/GFP population, having already given rise to OPCs.

To further determine the relationship of the identified populations, we developed an algorithm for single-cell near-neighbor network embedding and lineage determination (SCN3E, see STAR Methods for further details). By identifying the likely neighbors for each individual cell, we can infer which cells are most likely to be the progeny or progenitor of others. As a proof of concept, we analyzed the OL lineage populations identified in our previous single-cell RNA-seq work (Marques et al., 2016). SCN3E led to a similar ordering to the one obtained by t-SNE and Monocle, with a clear path from OPCs to MFOLs (Marques et al., 2016), but allowed a better resolution in the late differentiation and/or maturation, indicating a branching event at the MFOL stage (Figure S5B). SCN3E analysis for forebrain and spinal cord datasets indicated that NP1a cells (Figures 3D and 3E, arrows) were more related to post-natal OPCs, which then give rise to COPs and the remaining OL lineage. To verify our results using SCN3E, we used Monocle 2 (Qiu et al., 2017), an alternative ‘‘pseudo-time’’ algorithm, that orders cells in reconstructed pseudo-trajectories. Monocle2 generated similar results as SCN3E (Figures S6A and S6B).

A subset of NP1a cells expressed genes as *Olig1/2* and *Ptptrz1* but also Nestin (*Nes*) and *Bcan* (Figures 3D, 3E, and S4A), suggesting that they might constitute pre-OPCs. Monocle2 ordering of cells in a reconstructed pseudo-trajectory also revealed early OL lineage expression of *Ptptrz1*, *Bcan*, and *Olig2* (Figure S6B), comparable to the findings using our SCN3E pipeline. Interestingly, we also observed sparse expression in these cells of *Rfx4* (Figure 3D), which has been reported to be present in Sox10+ cells at E13.5 in the ventral midbrain, within a radial glia population, mRgl2 (La Manno et al., 2016). To further validate our findings, we applied SCN3E to this orthologous embryonic midbrain single-cell RNA-seq dataset (Figure S5C) (La Manno et al., 2016), and indeed found a sub-population within mRgl2 expressing *Bcan*, *Olig1*, *Ptprz1*, and lower levels of Nestin (Figure S5C), suggesting that this might constitute an NP1a population in the ventral midbrain. In sum, SCN3E analysis suggests that *Bcan*$^+$/ *Olig1/2*$^+$/*Ptptrz1*$^+$/*Nes*$^+$ NP1a cells are likely to be the progenitors of OPCs in the brain, constituting a pre-OPC cell state.

Bulk RNA-seq analysis showed that patterning and/or specification TFs were substantially down-regulated from E13.5 to P7, while TFs with roles in OL differentiation emerged (Figures 1B and 1C). We observed that *Hoxb3* and *Sox11* expression was also reduced in the transition from pre-OPCs to OPCs in the single-cell RNA-seq data (Figure 3E). We also observed such transitions when we ordered cells along SCN3E-derived pseudotime, selecting the 50 most significant variable genes along pseudo-time (Figures 3F and 3G). Indeed, several on-off transitions of cohort of genes occurred during different stages of the pseudo-time, with, for instance, genes expressed at NPs being down-regulated from the OPC stage on, being replaced by genes involved in differentiation and myelination at the COP/NFOL stage (Figures 3F and 3G). These transitions were observed both in the brain and spinal cord, highlighting transcriptional convergence between these two regions.

E13.5 *Pdgfra*+ Cells Give Rise Mainly to OPCs in the Post-natal CNS, but Also to Cells of the Pericyte Lineage

Our single-cell data indicated the presence of a subset of E13.5 *Pdgfra*/GFP+ cells possibly belonging to the pericyte lineage. To investigate whether these cells can indeed give rise to cells of this alternative lineage, we performed lineage-tracing experiments by injecting tamoxifen in E12.5 Pdgfra-Cre^{ERT}-LoxP-eGFP mice (Kang et al., 2010) and examined the GFP+ population at P21 (Figure 4A, see STAR Methods for more details). 47 ± 6% of the GFP + cells in the corpus callosum were OPCs (Pdgfra+/Col1−) (Figures 4B and 4E), while 15 ± 4% of E13.5 expressed Col1a1, a marker of the VLMC and pericyte lineages (Marques et al., 2016) (Figures 4D and 4E). Some of the remaining GFP+ populations were mature OLs (Figure 4C). Similar proportions were observed in the dorsal horn (spinal cord) (Figures 4B, 4C, and 4E). Thus, a subset of E13.5 *Pdgfra*+/GFP cells (most likely eVLMCs or PLCs) belong to the pericyte lineage and retain these properties or give rise to new cells of this lineage (e.g., pnVLMCs, VLMCs) at post-natal stages. Surprisingly, we found that at P21 there was still a considerable contribution of E13.5 *Pdgfra*+ cells to the OL lineage, and in particular to OPCs (Figures 4B, 4C, and 4E).

Forebrain *Nkx2.1*-expressing OL progenitors have been reported to originate at E12.5, but to have a minor contribution to dorsal forebrain OL lineage at post-natal stages (Tripathi et al., 2011; Kessaris et al., 2006). Since our data indicated that the first wave of oligodendrogenesis gives rise primarily to OPCs after birth, we examined the percentage of OPCs (Pdgfra+/Col1−) originating from this wave (GFP+ cells), compared to subsequent waves (GFP− cells). In the corpus callosum, less than 20% of OPCs were derived from the first wave, while the majority was derived from subsequent waves (Figure 4F). This is consistent with the findings that 20% of OL lineage cells (Sox10+) in the corpus callosum at P12–P13 are derived from the first and/or second wave, while 80% originate from the third one (Tripathi et al., 2011). In contrast, in the dorsal horn 48 ± 4% of the OPCs were derived from the E13.5 wave (Figure 4F). Thus, our results indicate that the progeny of the first wave of E13.5 *Pdgfra*+ progenitors persists in the juvenile CNS, with sub-populations giving rise not only to a specific population of OPCs but also to cells of other lineages, such as pericytes.

E13.5-Derived Post-natal OPCs and OPCs Derived from Subsequent Waves Have Similar Transcriptional Profiles and Electrophysiological Properties

Our single-cell transcriptomics analysis suggests that embryonic OL progenitors might undergo a process of rapid change of their transcriptional networks. Given that we observed that E13.5 *Pdgfra*/GFP+ progenitors give rise to a subset of the OPC population in the post-natal CNS (Figure 4), we investigated whether this convergence indeed occurs during development *in vivo*. We performed lineage tracing of *Pdgfra*+ cells originating from only the first wave (tamoxifen treatment at E12.5–txE12.5) or from all waves (tamoxifen treatment at P3–txP3) and assessed their progeny at P7 by single-cell RNA-seq (Figure 5A). We observed that OPCs derived from E13.5 or from subsequent waves intermingled (Figure 5B), with OPC1a/b and OPCcyc cells

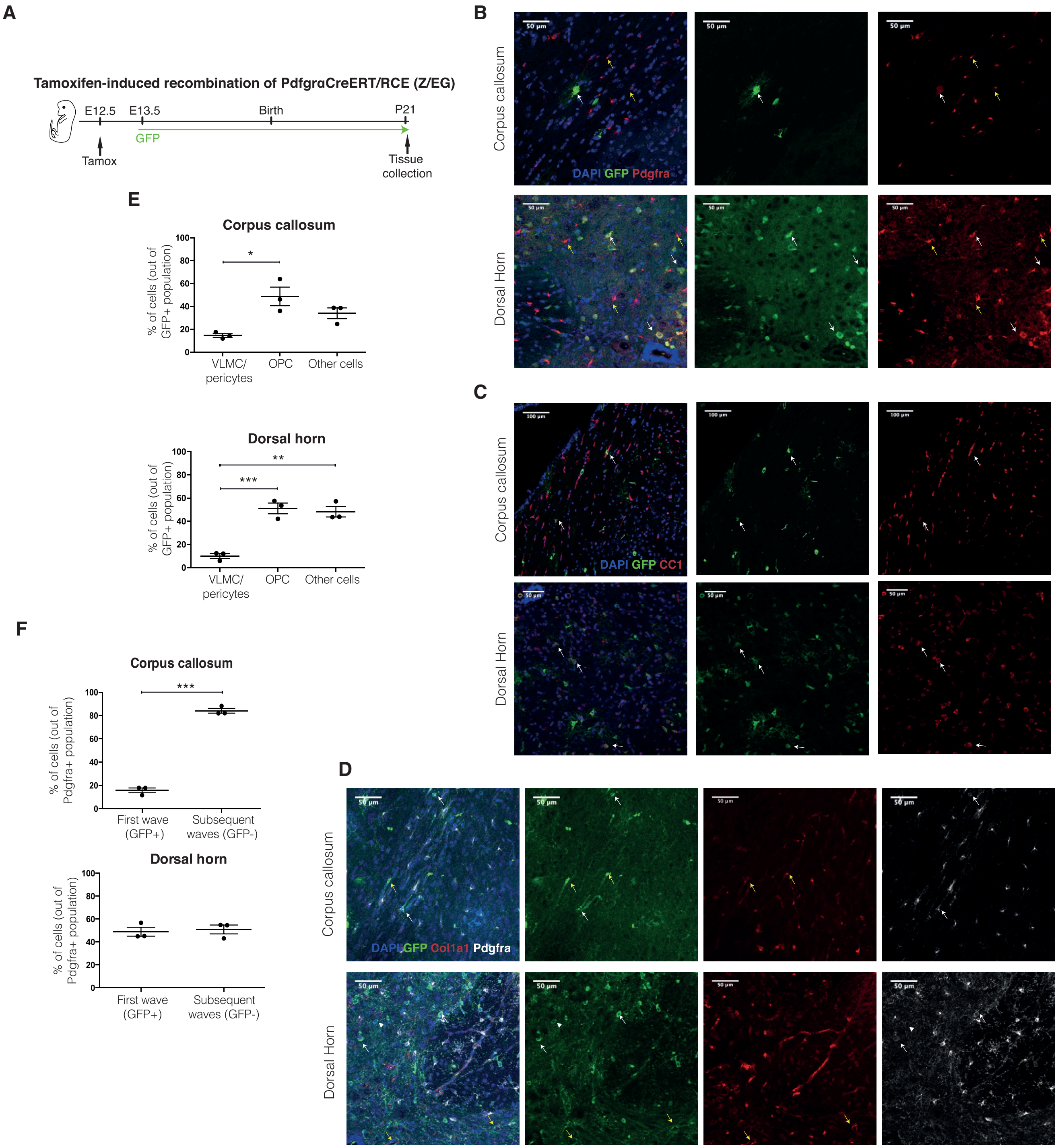

Figure 4. E13.5 *Pdgfra*+ Cells Give Rise to OPCs and Cells of the VLMC/Pericyte Lineage

(A) Scheme of lineage tracing experiments in Pdgfra-CreERT-RCE mice; E13.5 *Pdgfra*+ cells progeny was followed until P21 by GFP expression.

(B–D) Immunohistochemistry targeting Pdgfra+ cells (B and D), CC1+ mature oligodendrocytes (C) and cells of the VLMC/pericyte lineage (Col1a1+, D) in E13.5 Pdgfra+ progeny (GFP+) in corpus callosum and spinal cord (dorsal horn). B - White arrows, double positive Pdgfra/GFP cells. C - White arrows, double positive CC1/GFP cells. D -White arrows, double positive Pdgfra/GFP cells; yellow arrows, double positive Col1a1/GFP cells; arrowhead, GFP+/Pdgfra-/Col1a1- cell.

(E) Quantification of OPCs, VLMCs/pericytes, and other cells derived from E13.5 *Pdgfra*+ cells in the corpus callosum and dorsal horn; one-way ANOVA with Tukey's multiple comparisons test.

(F) Quantification of OPCs derived from the first wave (GFP+/ PDGFRa+) and subsequent waves (GFP−/ PDGFRa+) in the corpus callosum and dorsal horn. Two-tailed unpaired t test. All results are expressed as means ± SEM. For quantifications, 3 animals were used in each time point and 4–5 slices were photographed per animal. An average of 33 and 46 photos in CC and dorsal horn, respectively, were counted per animal. *, p value < 0.05; **, p value < 0.01; ***, p value < 0.001.

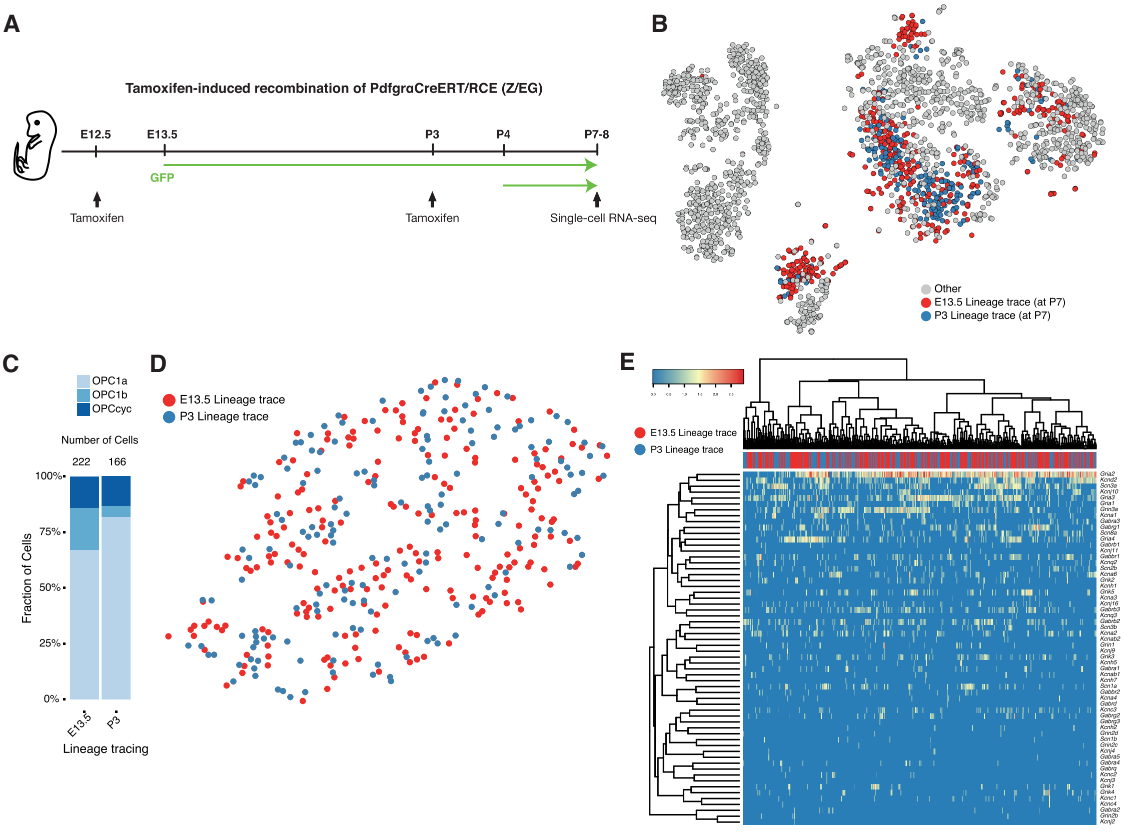

Figure 5. Similar Single-Cell Transcriptomic Profiles of Cells Derived from the First and Subsequent Waves of Oligodendrogenesis

(A) Scheme of lineage tracing experiments in Pdgfra-CreERT-RCE mice; E12.5-13.5 and P3-5 *Pdgfra*+ cells progeny was identified by GFP expression at P7-8 when single-cell RNA-seq was performed.

(B) t-SNE (from Figure 2) illustrating GFP+ cells from the lineage tracing of E12-5-13.5 or P3-P5 Pdgfra+ cells in the PdgfraCreERT-RCE mice at P7.

(C) Fraction of OPC1a, 1b and OPCcyc in each lineage tracing experiment.

(D) t-SNE clustered using glutamate receptor, potassium channel, voltage gated ion channel, and GABA receptor genes, illustrating homogeneous distribution of E13.5 and P3 lineage traced OPCs.

(E) Hierarchically clustered heatmap showing the expression of glutamate receptor, potassium channel, voltage-gated ion channel, and GABA receptor genes in E13.5 and P3 lineage traced OPCs.

being progeny of both the first wave and the subsequent waves (Figure 5C). OPC1b had a smaller representation in P3-derived OPCs, which might suggest that a defined temporal window is required for the maturation of *Pdgfra*/GFP+ into OPC1b, as well as pnVLMCs and COPs. We also analyzed the lineage-traced cells, focusing only on the expression profiles of genes involved in electrophysiological activity: glutamate receptor, potassium channel, voltage gated ion channel, and GABA receptor genes. t-SNE representation indicated that variation between these genes could not segregate txE12.5 from txP3 cells (Figure 5D), reflecting that these genes were expressed in a stochastic manner in both populations (Figure 5E). Thus, our results indicate that OPCs from different development temporal and spatial origins indeed converge into three transcriptional states at post-natal stages.

OPCs have been shown to present electrophysiological properties (Clarke et al., 2012; Káradóttir et al., 2008), and our single-cell RNA-seq experiments coupled with lineage tracing would suggest that E13.5-derived OPCs at P7 have similar electrophysiological properties to OPCs derived from subsequent waves (Figure 5E). In order to investigate if this is indeed the case, we performed whole-cell voltage-clamp recordings of corpus callosum OPCs at P7-P8 derived from either the first wave (tamoxifen treatment at E12.5–txE12.5) or all waves (tamoxifen treatment at P3–txP3) (Figures 6A and 6B). The two groups (slices from 2 animals txE12.5 and from 4 animals txP3) exhibited comparable passive intrinsic properties: input resistance (txE12.5 1.6 ± 0.2 GΩ; txP3 1.6 ± 0.2 GΩ; (n) of cells = 6 and 15, respectively), and membrane capacitance (txE12.5: 34.3 ± 8.7 pF, n = 7; txP3: 30.7 ± 6.1 pF, n = 15). Recorded events had similar average amplitudes (txE12.5: −12.28 ± 0.39 pA, n = 7; txP3: −13.0 ± 0.4 pA, n = 15). The frequency of events in the two populations was also not significantly different (Figures 6C and 6D). However, while txE12.5 cells received less frequent events in a more uniform manner (6 ± 1.89 Hz, n = 7), txP3 cells exhibited a larger heterogeneity, with 30% of the cells having more frequent events (11.4 ± 3.1 Hz, n = 15; p = 0.3). Concurrently, the cumulative distribution of event amplitudes displayed somewhat

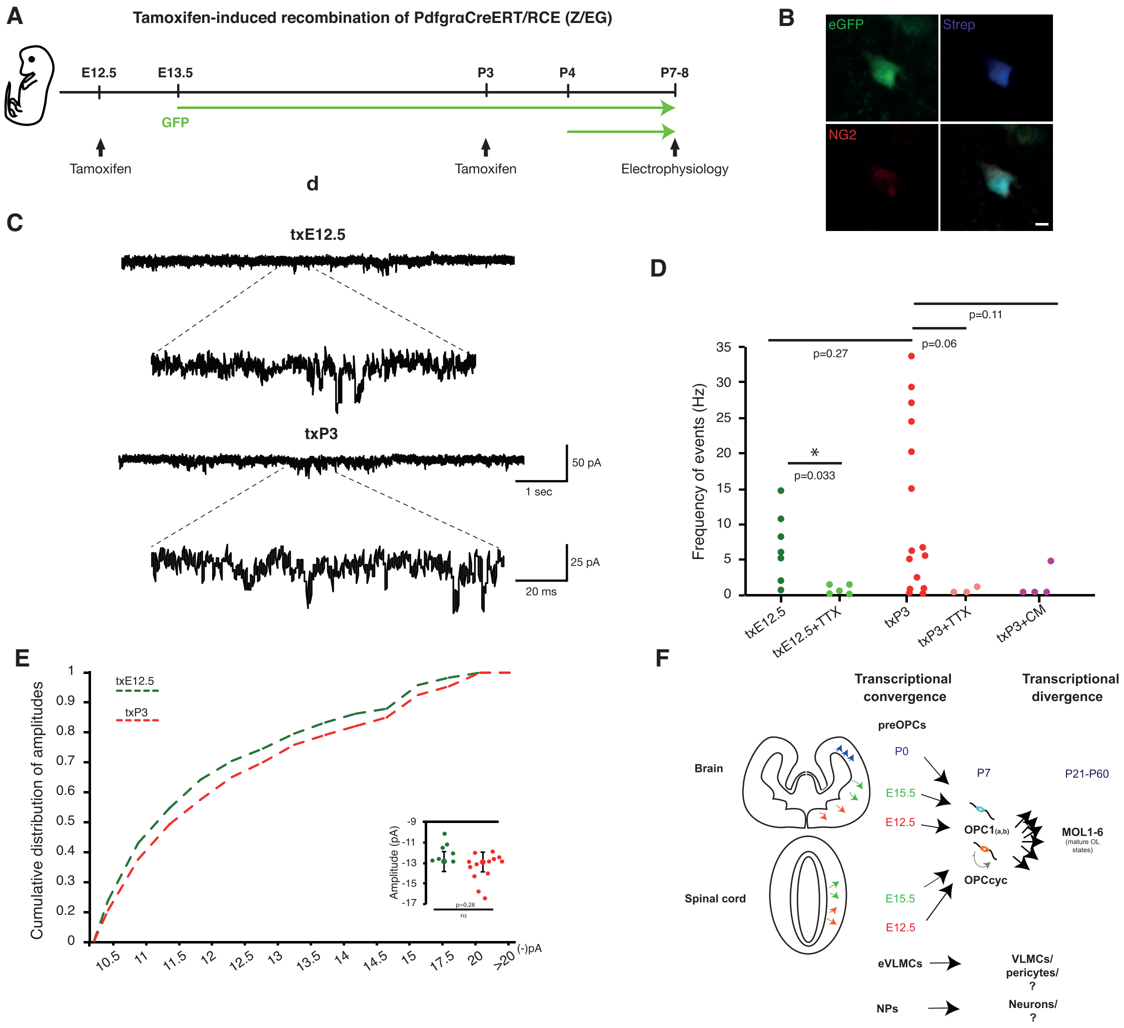

Figure 6. Similar Electrophysiological Profiles of Cells Derived from the First and Subsequent Waves of Oligodendrogenesis

(A) Scheme of lineage tracing experiments in Pdgfra-CreERT-RCE mice; E12.5-13.5 and P3-5 *Pdgfra+* cells progeny was identified by GFP expression at P7-8 when electrophysiological recordings were performed.

(B) Representative image showing a recorded cell labeled with biocytin-streptavidin (blue) and expression of NG2 (red); Scale bar represents 5 μm.

(C) Representative voltage-clamp traces of OPCs (held at −70 mV) showing inward spontaneous post-synaptic currents.

(D) Frequency of events in seven txE12.5-cells (dark green) and fifteen txP3-cells (red). Tetrodotoxin (TTX) strongly reduced frequency spontaneous of events, shown as txE12.5+TTX (five cells) and txP3+TTX (three cells). Blockage of glutamatergic receptors with CNQX/MK801 showed similar effect (txP3-CM, four cells).

(E) Cumulative distribution of events' amplitudes and on the right side insert with average amplitudes for each cell. p values correspond to two-tailed Student's t test for independent (between groups) or paired-samples (for pharmacology experiments).

(F) Model for transcriptional convergence of the different waves of progenitors of oligodendrocyte lineage cells.

larger events recorded on OPCs derived from txP3 (Figure 6E). In order to confirm that these events are due to spiking activity in neighboring neurons, we blocked excitation by applying 10 μM of Tetrodotoxin (TTX) in slices from 2 animals per time point. This drastically reduced the frequency of events in both groups (Figure 6D): txE12+TTX (n = 5; 0.93 ± 0.33 Hz; p = 0.038) and txP3+TTX (n = 3; 0.54 ± 0.32 Hz; p = 0.075). Accordingly, when glutamatergic receptors were blocked, we also observed a robust decrease in number of events (txP3+CM−1.2 ± 1.3 Hz; n = 4; p = 0.11, Figure 6D). As such, OPCs derived from the E13.5 wave present similar electrophysiological properties to OPCs derived from subsequent waves.

DISCUSSION

We have previously shown that OL lineage in the juvenile and adult CNS is more heterogeneous than previously anticipated, with several intermediate states after differentiation of OPCs and six mature OL states (Marques et al., 2016). Here, we provide evidence that progenitor cells of the OL lineage with different temporal and spatial origins in the CNS converge into similar OPC transcriptional states at P7 (Figure 6F). Our results from bulk and single-cell RNA-seq suggest that a transcriptional network of embryonic patterning TFs is replaced at post-natal stages in the anterior and posterior CNS by a convergent

transcriptional network, associated with electrophysiological responses and compatible with activity-driven differentiation and myelination (Koudelka et al., 2016; Gautier et al., 2015; Wake et al., 2015; Gibson et al., 2014; Lundgaard et al., 2013; Demerens et al., 1996). Given the occurrence of this convergence into similar OPC states at post-natal stages, when differentiation starts, subsequent cell-state diversification into six mature OL cell states (Marques et al., 2016) is thus likely not cell intrinsic but rather induced by the local environment OPCs are exposed to upon differentiation (Figure 6F).

Previous transcriptomics studies on the OL lineage were based on bulk populations expressing *Pdgfra* and focused on single regions, such as the forebrain (Moyon et al., 2015; Zhang et al., 2014; Cahoy et al., 2008). These studies were performed with bulk RNA-seq or microarrays, and suggested, for instance, that neonatal and adult OPCs presented different transcriptional profiles (Moyon et al., 2015). In our study, bulk and single-cell RNA transcriptomics were, for the first time, performed on the exact same *Pdgfra*+ cell population at different stages and regions, giving an unbiased and clear understanding of what exactly these cells are at the molecular level and how diverse the *Pdgfra*+ population is. Bulk RNA-seq analysis of FACS-sorted *Pdgfra*+/GFP cells suggests heterogeneity between P7 OPCs from the spinal cord and brain, with the former expressing genes involved in myelination at substantially higher levels. However, scRNA-seq analysis of the populations present at this stage indicates that OPCs are unexpectedly similar at the transcriptional level, highlighting the strength of single-cell RNA-seq analysis to deconvolute bulk transcriptomics analysis.

OPCs in different anterior-posterior regions of the CNS converge into similar transcriptional profiles at P7 that are compatible to their main functions at that stage (integration of neuronal activity and differentiation). Nevertheless, it is possible that small cohorts of differentially expressed genes (Table S3) might be involved in additional functions of OPCs, contributing to different functional states within the OPC cell type within the different regions or stages. Alternatively, it is possible that intrinsic epigenetic differences between brain and spinal cord OPCs are dormant, not reflected at a transcriptional level. Such epigenetic memory, possibly in the form of post-translational modifications at the level of histones, could then be reactivated upon specific environmental stimuli upon differentiation. Further epigenomic experiments may elucidate whether this is indeed the case.

Our data indicate that only a subset of *Pdgfra*/GFP+ cells at E13.5 express markers of the future OL lineage, and that other subsets have transcriptional profiles compatible with progenitors of other cell lineages. Lineage tracing had previously indicated that a small subset of post-natal *Pdgfra*/GFP+ cells can give rise to neurons, although it was not clear whether this was a technical artifact due to spurious Cre recombination (Clarke et al., 2012). Motor neurons are specified at E9-10.5 at the ventral CNS from an Olig2 domain, while a subsequent wave of oligodendrogenesis starts occurring at E12.5, with the emergence of *Pdgfra*+ cells (Rowitch, 2004). Thus, oligodendrogenesis, rather than neurogenesis, should be captured from this domain at E13.5. Since we performed lineage tracing with Pdgfra-Cre-ERT/RCE mice upon injection of tamoxifen at E12.5/13.5 and P3/P4 and combined with single-cell RNA-Seq at P7/8, we could investigate whether this population can give rise to neurons. Correlation analysis of this dataset with a previously published single-cell RNA-seq dataset (Zeisel et al., 2015) indicates that the lineage-traced cells show high correlation with OLs and astrocytes and/or ependymal cells, and much lower with microglia, endothelial-mural cells or neurons (Figure S6C). Nevertheless, four E13.5-derived cells have a high correlation with neurons (Figure S6C). Additional single-cell RNA-seq will be required to elucidate whether embryonic *Pdgfra*/GFP+ cells indeed can give rise to neurons, since it is possible that the four cells observed are due to spurious recombination.

The identification of progenitors of cells of the pericyte lineage has been elusive (Armulik et al., 2011). We observed E13.5 *Pdgfra*+ populations that express pericyte and VLMC markers and can give rise to cells of the pericyte lineage. Pericytes and OL progenitors have been described to share antigenic properties, including expression of *Pdgfra* and *Cspg4* (NG2). Adult *Pdgfra*+ cells have also been described to occasionally give rise to pericytes (Kang et al., 2010). Thus, it is possible that *Pdgfra*+ eVMLC, or even one of the NP populations, are the progenitors of at least a subset of cells in the pericyte lineage. Interestingly, it has been previously reported that E7.5 Sox10+ neural crest cells give rise to pericytes in the adult CNS (Simon et al., 2012). While we do not detect Sox10 expression in eVLMCs or NPs, it is possible that these cells are derived from an earlier progenitor population expressing *Sox10*. pnVLMCs, in contrast to pericytes and eVLMC, express several genes that are present in cells of the OL lineage, such as *Plp1*, *Mbp*, and *Trf*. This could suggest a lineage relationship with OLs. Nevertheless, SC3NE and Monocle 2 (Figures 3 and S6) indicate that such a link is unlikely. As such, we consider that the most likely explanation of our data is that distinct sub-populations of *Pdgfra*+ cells at E13.5 give rise to different lineages, rather than the existence of a multipotential *Pdgfra*+ progenitor that gives rise both to OLs and pericytes. These might have been generated from the *Pdgfra*+ NPs described above, or from a separate source, as cells associated with blood vessels invading the developing CNS. Lineage tracing using eVLMC or NP specific markers might elucidate whether indeed any of these populations are progenitors within the pericyte lineage.

OPCs derived from the E13.5 wave presented similar electrophysiological properties to OPCs derived from subsequent waves (Figure 6). Nevertheless, there was a bimodal pattern of electrophysiological activity for P3-derived OPCs. A subset of second and/or third-wave-derived OPCs are more responsive to the neighboring neuronal network than E13.5-derived OPCs (Figure 6D), suggesting a higher heterogeneity of second and/or third-wave-derived OPCs than first-wave-derived OPCs. When analyzing the overall transcriptional profile of OPCs derived from the E13.5 wave and other waves, no heterogeneity was found (Figure 5), indicating that the transcriptional state could thus not account for the observed electrophysiological differences. We observed scattered expression of several ion channels and glutamate receptors (Figure 5E), which can be consistent with cell heterogeneity at an electrophysiological level. Techniques such as Patch-Seq (Fuzik et al., 2016) might be able to address if this is indeed the case. Alternatively,

other post-transcriptional events, such as asymmetric distribution of transcripts in OPC processes (Thakurela et al., 2016) or translational events, might account for the observed differences.

Our unexpected finding that post-natal OPCs from different regions are transcriptionally similar suggests a strong selective pressure during development to assure functional convergence of these progenitor populations. This is in contrast with neuronal lineages, where diversity of cell states arises early during development, with progenitors expressing patterning TFs that will ultimately determine the identity of their progeny (Mayer et al., 2018; Mi et al., 2018). Interestingly, many of these TFs are actively down-regulated upon terminal neuronal differentiation, while their ectopic reactivation has been linked with cell death and degeneration (von Schimmelmann et al., 2016). Nevertheless, the attenuation of patterning and/or specification transcription we observe in the OL lineage is not a common event during neural development. For instance, it does not occur within the dopaminergic neuronal lineage, where patterning TFs such as Lmx1a/b and Nurr1 continue to be expressed at adult stages and have distinct functions relative to their developmental ones (Doucet-Beaupré et al., 2016; Kadkhodaei et al., 2013). Our results indicate that down-regulation of patterning and specification TFs appear to be required for the establishment of an OPC transcriptional state. Abnormal re-expression or retainment of TFs expressed during embryogenesis might be deleterious to post-natal OPCs or might prevent their capacity to differentiate in the context of disease.

STAR★METHODS

Detailed methods are provided in the online version of this paper and include the following:

- KEY RESOURCES TABLE
- CONTACT FOR REAGENT AND RESOURCE SHARING
- EXPERIMENTAL MODEL AND SUBJECT DETAILS
  - Mice
- METHOD DETAILS
  - OPC Extraction
  - FACS Sorting
  - RNA Extraction for Bulk RNA-Seq
  - Library Preparation for Bulk RNA-Seq
  - Single-Cell RNA-Seq Cell Capture and Imaging
  - Lineage Tracing
  - Electrophysiology
  - Immunohistochemistry
  - Microscopy
- QUANTIFICATION AND STATISTICAL ANALYSIS
  - Statistical Analysis of Gene Expression of Bulk RNA-Seq
  - Gene Ontology
  - Single-Cell RNA-Seq
  - Single-Cell Clustering
  - BackSPIN Clustering
  - Pathway and Geneset Overdispersion Analysis (PAGODA)
  - Unbiased Cluster Determination PAGODA
  - Merging of BackSPIN2 and PAGODA Clusters
  - Single-Cell Near-Neighbor Network Embedding (SCN3E)
  - Differential Gene Expression
  - Electrophysiology Analysis
  - Microscopy Analysis
- DATA AND SOFTWARE AVAILABILITY
  - Data Resources
  - Software
- ADDITIONAL RESOURCES

SUPPLEMENTAL INFORMATION

Supplemental Information includes six figures and four tables and can be found with this article online at https://doi.org/10.1016/j.devcel.2018.07.005.

ACKNOWLEDGMENTS

We would like to thank Amit Zeisel, Tony Jimenez-Beristain, Alessandra Nanni, Ahmad Moshref, and Johnny Söderlund, and the Single Cell Genomics Facility and Wallenberg Advanced Bioinformatics Infrastructure (WABI) Long Term Bioinformatic Support at SciLifeLab for support. The bioinformatics computations were performed on resources provided by the Swedish National Infrastructure for Computing (SNIC) at UPPMAX, Uppsala University. D.V. would like to acknowledge the University of Sydney HPC service at The University of Sydney for providing HPC resources that have contributed to the research reported in this paper. This work was supported, in part, by a University of Sydney HPC Grand Challenge Award. D.V. was supported in part by a Boehringer Ingelheim Travel Grant. Work in RJT lab was supported by Australian NHMRC grant APP1043023. L.V., S.G., and Å.B. are supported by a grant from the Knut and Alice Wallenberg Foundation to WABI. J.H.-L. is supported by VR, StratNeuro, Hjärnfonden, and EU FP7/Marie Curie Actions. A.M.F. is supported by the European Committee for Treatment and Research of Multiple Sclerosis (ECTRIMS). Work in G.C.-B.'s research group was supported by Swedish Research Council (grants 2010-3114 and 2015-03558), European Union (FP7/Marie Curie Integration Grant EPIOPC grant agreement number 333713, Horizon 2020 Research and Innovation Programme/European Research Council Consolidator Grant EPIScOPE, grant agreement number 681893), Swedish Brain Foundation (Hjärnfonden, grant FO2015-0182), Ming Wai Lau Centre for Reparative Medicine, Swedish Cancer Society (Cancerfonden, grant CAN 2016/555), Petrus och Augusta Hedlunds Foundation (grants M-2014-0041 and M-2016-0428), and Karolinska Institutet. A web resource for browsing differential gene expression data for the single-cell and bulk data can be accessed at our resource webpage https://ki.se/en/mbb/oligointernode.

AUTHOR CONTRIBUTIONS

S.M., D.v.B., D.V., and G.C.-B. designed all experiments. S.M. and D.v.B. performed and D.v.B., L.V., S.G., and Å.K.B. analyzed the single-cell RNA-seq data. S.M. and D.V. performed and D.V. analyzed the bulk RNA-seq data, which R.J.T. oversaw. S.M., E.M.F., H.M., A.M.F., and M.M. performed all other experiments. J.H.-L. oversaw electrophysiology experiments. S.M., D.V., D.v.B. and G.C.-B. wrote the manuscript with input from the co-authors. G.C.B. oversaw all aspects of the study.

DECLARATION OF INTERESTS

The G.C.-B. research group has received funding from F. Hoffmann – La Roche, Ltd. for an unrelated research project. R.J.T. is an employee of Illumina, Inc.

Received: September 5, 2017
Revised: May 23, 2018
Accepted: July 3, 2018
Published: August 2, 2018

REFERENCES

Alexa, A., Rahnenführer, J., and Lengauer, T. (2006). Improved scoring of functional groups from gene expression data by decorrelating GO graph structure. Bioinformatics *22*, 1600–1607.

Alexa, A.R., and Rahnenfuhrer, J. (2016). topGO: Enrichment Analysis for Gene Ontology. R Package Version 2.26.0 (R Foundation for Statistical Computing).

Andrews, S. (2010) FastQC: a quality control tool for high throughput sequence data, http://www.bioinformatics.babraham.ac.uk/projects/fastqc.

Armulik, A., Genové, G., and Betsholtz, C. (2011). Pericytes: developmental, physiological, and pathological perspectives, problems, and promises. Dev. Cell *21*, 193–215.

Bechler, M.E., Byrne, L., and Ffrench-Constant, C. (2015). CNS myelin sheath lengths are an intrinsic property of oligodendrocytes. Curr. Biol. *25*, 2411–2416.

Buettner, F., Pratanwanich, N., McCarthy, D.J., Marioni, J.C., and Stegle, O. (2017). f-scLVM: scalable and versatile factor analysis for single-cell RNA-seq. Genome Biol. *18*, 212.

Butt, S.J., Sousa, V.H., Fuccillo, M.V., Hjerling-Leffler, J., Miyoshi, G., Kimura, S., and Fishell, G. (2008). The requirement of Nkx2-1 in the temporal specification of cortical interneuron subtypes. Neuron *59*, 722–732.

Cahoy, J.D., Emery, B., Kaushal, A., Foo, L.C., Zamanian, J.L., Christopherson, K.S., Xing, Y., Lubischer, J.L., Krieg, P.A., Krupenko, S.A., et al. (2008). A transcriptome database for astrocytes, neurons, and oligodendrocytes: a new resource for understanding brain development and function. J. Neurosci. *28*, 264–278.

Cai, J., Qi, Y., Hu, X., Tan, M., Liu, Z., Zhang, J., Li, Q., Sander, M., and Qiu, M. (2005). Generation of oligodendrocyte precursor cells from mouse dorsal spinal cord independent of Nkx6 regulation and Shh signaling. Neuron *45*, 41–53.

Clarke, L.E., Young, K.M., Hamilton, N.B., Li, H., Richardson, W.D., and Attwell, D. (2012). Properties and fate of oligodendrocyte progenitor cells in the corpus callosum, motor cortex, and piriform cortex of the mouse. J. Neurosci. *32*, 8173–8185.

Cline, M.S., Smoot, M., Cerami, E., Kuchinsky, A., Landys, N., Workman, C., Christmas, R., Avila-Campilo, I., Creech, M., Gross, B., et al. (2007). Integration of biological networks and gene expression data using cytoscape. Nat. Protoc. *2*, 2366–2382.

Coffey, J.C., and McDermott, K.W. (1997). The regional distribution of myelin oligodendrocyte glycoprotein (MOG) in the developing rat CNS: an in vivo immunohistochemical study. J. Neurocytol. *26*, 149–161.

Conesa, A., Madrigal, P., Tarazona, S., Gomez-Cabrero, D., Cervera, A., McPherson, A., Szcześniak, M.W., Gaffney, D.J., Elo, L.L., Zhang, X., et al. (2016). A survey of best practices for RNA-seq data analysis. Genome Biol. *17*, 13.

Crawford, A.H., Tripathi, R.B., Richardson, W.D., and Franklin, R.J.M. (2016). Developmental origin of oligodendrocyte lineage cells determines response to demyelination and susceptibility to age-associated functional decline. Cell Rep. *15*, 761–773.

Demerens, C., Stankoff, B., Logak, M., Anglade, P., Allinquant, B., Couraud, F., Zalc, B., and Lubetzki, C. (1996). Induction of myelination in the central nervous system by electrical activity. Proc. Natl. Acad. Sci. USA *93*, 9887–9892.

Dobin, A., Davis, C.A., Schlesinger, F., Drenkow, J., Zaleski, C., Jha, S., Batut, P., Chaisson, M., and Gingeras, T.R. (2013). STAR: ultrafast universal RNA-seq aligner. Bioinformatics *29*, 15–21.

Doucet-Beaupré, H., Gilbert, C., Profes, M.S., Chabrat, A., Pacelli, C., Giguère, N., Rioux, V., Charest, J., Deng, Q., Laguna, A., et al. (2016). Lmx1a and Lmx1b regulate mitochondrial functions and survival of adult midbrain dopaminergic neurons. Proc. Natl. Acad. Sci. USA *113*, E4387–E4396.

Durinck, S., Spellman, P.T., Birney, E., and Huber, W. (2009). Mapping identifiers for the integration of genomic datasets with the R/Bioconductor package biomaRt. Nat. Protoc. *4*, 1184–1191.

Fan, J., Salathia, N., Liu, R., Kaeser, G.E., Yung, Y.C., Herman, J.L., Kaper, F., Fan, J.B., Zhang, K., Chun, J., et al. (2016). Characterizing transcriptional heterogeneity through pathway and gene set overdispersion analysis. Nat. Methods *13*, 241–244.

Finak, G., McDavid, A., Yajima, M., Deng, J., Gersuk, V., Shalek, A.K., Slichter, C.K., Miller, H.W., McElrath, M.J., Prlic, M., et al. (2015). MAST: a flexible statistical framework for assessing transcriptional changes and characterizing heterogeneity in single-cell RNA sequencing data. Genome Biol. *16*, 278.

Fogarty, M., Richardson, W.D., and Kessaris, N. (2005). A subset of oligodendrocytes generated from radial glia in the dorsal spinal cord. Development *132*, 1951–1959.

Fuzik, J., Zeisel, A., Máté, Z., Calvigioni, D., Yanagawa, Y., Szabó, G., Linnarsson, S., and Harkany, T. (2016). Integration of electrophysiological recordings with single-cell RNA-seq data identifies neuronal subtypes. Nat. Biotechnol. *34*, 175–183.

Gautier, H.O., Evans, K.A., Volbracht, K., James, R., Sitnikov, S., Lundgaard, I., James, F., Lao-Peregrin, C., Reynolds, R., Franklin, R.J., et al. (2015). Neuronal activity regulates remyelination via glutamate signalling to oligodendrocyte progenitors. Nat. Commun. *6*, 8518.

Gentleman, R.C., Carey, V.J., Bates, D.M., Bolstad, B., Dettling, M., Dudoit, S., Ellis, B., Gautier, L., Ge, Y., Gentry, J., et al. (2004). Bioconductor: open software development for computational biology and bioinformatics. Genome Biol. *5*, R80.

Gibson, E.M., Purger, D., Mount, C.W., Goldstein, A.K., Lin, G.L., Wood, L.S., Inema, I., Miller, S.E., Bieri, G., Zuchero, J.B., et al. (2014). Neuronal activity promotes oligodendrogenesis and adaptive myelination in the mammalian brain. Science *344*, 1252304.

Haghverdi, L., Büttner, M., Wolf, F.A., Buettner, F., and Theis, F.J. (2016). Diffusion pseudotime robustly reconstructs lineage branching. Nat. Methods *13*, 845–848.

Hart, S.N., Therneau, T.M., Zhang, Y., Poland, G.A., and Kocher, J.P. (2013). Calculating sample size estimates for RNA sequencing data. J. Comput. Biol. *20*, 970–978.

Hirano, M., and Goldman, J.E. (1988). Gliogenesis in rat spinal cord: evidence for origin of astrocytes and oligodendrocytes from radial precursors. J. Neurosci. Res. *21*, 155–167.

Hrvatin, S., Hochbaum, D.R., Nagy, M.A., Cicconet, M., Robertson, K., Cheadle, L., Zilionis, R., Ratner, A., Borges-Monroy, R., Klein, A.M., et al. (2018). Single-cell analysis of experience-dependent transcriptomic states in the mouse visual cortex. Nat. Neurosci. *21*, 120–129.

Islam, S., Zeisel, A., Joost, S., La Manno, G., Zajac, P., Kasper, M., Lönnerberg, P., and Linnarsson, S. (2014). Quantitative single-cell RNA-seq with unique molecular identifiers. Nat. Methods *11*, 163–166.

Jang, E., Lee, S., Kim, J.H., Kim, J.H., Seo, J.W., Lee, W.H., Mori, K., Nakao, K., and Suk, K. (2013). Secreted protein lipocalin-2 promotes microglial M1 polarization. FASEB J. *27*, 1176–1190.

Kadkhodaei, B., Alvarsson, A., Schintu, N., Ramsköld, D., Volakakis, N., Joodmardi, E., Yoshitake, T., Kehr, J., Decressac, M., Björklund, A., et al. (2013). Transcription factor Nurr1 maintains fiber integrity and nuclear-encoded mitochondrial gene expression in dopamine neurons. Proc. Natl. Acad. Sci. USA *110*, 2360–2365.

Kang, S.H., Fukaya, M., Yang, J.K., Rothstein, J.D., and Bergles, D.E. (2010). NG2+ CNS glial progenitors remain committed to the oligodendrocyte lineage in postnatal life and following neurodegeneration. Neuron *68*, 668–681.

Káradóttir, R., Hamilton, N.B., Bakiri, Y., and Attwell, D. (2008). Spiking and nonspiking classes of oligodendrocyte precursor glia in CNS white matter. Nat. Neurosci. *11*, 450–456.

Kessaris, N., Fogarty, M., Iannarelli, P., Grist, M., Wegner, M., and Richardson, W.D. (2006). Competing waves of oligodendrocytes in the forebrain and postnatal elimination of an embryonic lineage. Nat. Neurosci. *9*, 173–179.

Kim, D., Langmead, B., and Salzberg, S.L. (2015). HISAT: a fast spliced aligner with low memory requirements. Nat. Methods *12*, 357–360.

Klinghoffer, R.A., Hamilton, T.G., Hoch, R., and Soriano, P. (2002). An allelic series at the PDGFalphaR locus indicates unequal contributions of distinct signaling pathways during development. Dev. Cell *2*, 103–113.

Koudelka, S., Voas, M.G., Almeida, R.G., Baraban, M., Soetaert, J., Meyer, M.P., Talbot, W.S., and Lyons, D.A. (2016). Individual neuronal subtypes exhibit diversity in CNS myelination mediated by synaptic vesicle release. Curr. Biol. *26*, 1447–1455.

La Manno, G., Gyllborg, D., Codeluppi, S., Nishimura, K., Salto, C., Zeisel, A., Borm, L.E., Stott, S.R.W., Toledo, E.M., Villaescusa, J.C., et al. (2016). Molecular diversity of midbrain development in mouse, human, and stem cells. Cell *167*, 566–580.e19.

Larson, V.A., Mironova, Y., Vanderpool, K.G., Waisman, A., Rash, J.E., Agarwal, A., and Bergles, D.E. (2018). Oligodendrocytes control potassium accumulation in white matter and seizure susceptibility. Elife *7*, https://doi.org/10.7554/eLife.34829.

Law, C.W., Chen, Y., Shi, W., and Smyth, G.K. (2014). Voom: precision weights unlock linear model analysis tools for RNA-seq read counts. Genome Biol. *15*, R29.

Liao, Y., Smyth, G.K., and Shi, W. (2014). featureCounts: an efficient general purpose program for assigning sequence reads to genomic features. Bioinformatics *30*, 923–930.

Love, M.I., Huber, W., and Anders, S. (2014). Moderated estimation of fold change and dispersion for RNA-seq data with DESeq2. Genome Biol. *15*, 550.

Lundgaard, I., Luzhynskaya, A., Stockley, J.H., Wang, Z., Evans, K.A., Swire, M., Volbracht, K., Gautier, H.O., Franklin, R.J., Charles Ffrench-Constant, et al. (2013). Neuregulin and BDNF induce a switch to NMDA receptor-dependent myelination by oligodendrocytes. PLOS Biol. *11*, e1001743.

Marques, S., Zeisel, A., Codeluppi, S., van Bruggen, D., Mendanha Falcão, A.M., Xiao, L., Li, H., Häring, M., Hochgerner, H., Romanov, R.A., et al. (2016). Oligodendrocyte heterogeneity in the mouse juvenile and adult central nervous system. Science *352*, 1326–1329.

Mayer, C., Hafemeister, C., Bandler, R.C., Machold, R., Batista Brito, R., Jaglin, X., Allaway, K., Butler, A., Fishell, G., and Satija, R. (2018). Developmental diversification of cortical inhibitory interneurons. Nature *555*, 457–462.

Merico, D., Isserlin, R., Stueker, O., Emili, A., and Bader, G.D. (2010). Enrichment map: a network-based method for gene-set enrichment visualization and interpretation. PLoS One *5*, e13984.

Mi, D., Li, Z., Lim, L., Li, M., Moissidis, M., Yang, Y., Gao, T., Hu, T.X., Pratt, T., Price, D.J., et al. (2018). Early emergence of cortical interneuron diversity in the mouse embryo. Science *360*, 81–85.

Micu, I., Plemel, J.R., Lachance, C., Proft, J., Jansen, A.J., Cummins, K., van Minnen, J., and Stys, P.K. (2016). The molecular physiology of the axo-myelinic synapse. Exp. Neurol. *276*, 41–50.

Moyon, S., Dubessy, A.L., Aigrot, M.S., Trotter, M., Huang, J.K., Dauphinot, L., Potier, M.C., Kerninon, C., Melik Parsadaniantz, S., Franklin, R.J., et al. (2015). Demyelination causes adult CNS progenitors to revert to an immature state and express immune cues that support their migration. J. Neurosci. *35*, 4–20.

Mudge, J.M., and Harrow, J. (2015). Creating reference gene annotation for the mouse C57BL6/J genome assembly. Mamm. Genome *26*, 366–378.

Nave, K.A., and Werner, H.B. (2014). Myelination of the nervous system: mechanisms and functions. Annu. Rev. Cell Dev. Biol. *30*, 503–533.

Pertea, M., Pertea, G.M., Antonescu, C.M., Chang, T.C., Mendell, J.T., and Salzberg, S.L. (2015). StringTie enables improved reconstruction of a transcriptome from RNA-seq reads. Nat. Biotechnol. *33*, 290–295.

Pringle, N.P., and Richardson, W.D. (1993). A singularity of PDGF alpha-receptor expression in the dorsoventral axis of the neural tube may define the origin of the oligodendrocyte lineage. Development *117*, 525–533.

Qiu, X., Mao, Q., Tang, Y., Wang, L., Chawla, R., Pliner, H.A., and Trapnell, C. (2017). Reversed graph embedding resolves complex single-cell trajectories. Nat. Methods *14*, 979–982.

Richardson, W.D., Smith, H.K., Sun, T., Pringle, N.P., Hall, A., and Woodruff, R. (2000). Oligodendrocyte lineage and the motor neuron connection. Glia *29*, 136–142.

Ritchie, M.E., Phipson, B., Wu, D., Hu, Y., Law, C.W., Shi, W., and Smyth, G.K. (2015). Limma powers differential expression analyses for RNA-sequencing and microarray studies. Nucleic Acids Res. *43*, e47.

Romanov, R.A., Zeisel, A., Bakker, J., Girach, F., Hellysaz, A., Tomer, R., Alpár, A., Mulder, J., Clotman, F., Keimpema, E., et al. (2017). Molecular interrogation of hypothalamic organization reveals distinct dopamine neuronal subtypes. Nat. Neurosci. *20*, 176–188.

Rowitch, D.H. (2004). Glial specification in the vertebrate neural tube. Nat. Rev. Neurosci. *5*, 409–419.

Simon, C., Lickert, H., Götz, M., and Dimou, L. (2012). Sox10-iCreERT2: a mouse line to inducibly trace the neural crest and oligodendrocyte lineage. Genesis *50*, 506–515.

Spassky, N., Olivier, C., Perez-Villegas, E., Goujet-Zalc, C., Martinez, S., Thomas, J.I., and Zalc, B. (2000). Single or multiple oligodendroglial lineages: a controversy. Glia *29*, 143–148.

Thakurela, S., Garding, A., Jung, R.B., Müller, C., Goebbels, S., White, R., Werner, H.B., and Tiwari, V.K. (2016). The transcriptome of mouse central nervous system myelin. Sci. Rep. *6*, 25828.

Tripathi, R.B., Clarke, L.E., Burzomato, V., Kessaris, N., Anderson, P.N., Attwell, D., and Richardson, W.D. (2011). Dorsally and ventrally derived oligodendrocytes have similar electrical properties but myelinate preferred tracts. J. Neurosci. *31*, 6809–6819.

Tsafrir, D., Tsafrir, I., Ein-Dor, L., Zuk, O., Notterman, D.A., and Domany, E. (2005). Sorting points into neighborhoods (SPIN): data analysis and visualization by ordering distance matrices. Bioinformatics *21*, 2301–2308.

Vallstedt, A., Klos, J.M., and Ericson, J. (2005). Multiple dorsoventral origins of oligodendrocyte generation in the spinal cord and hindbrain. Neuron *45*, 55–67.

van den Brink, S.C., Sage, F., Vértesy, Á., Spanjaard, B., Peterson-Maduro, J., Baron, C.S., Robin, C., and van Oudenaarden, A. (2017). Single-cell sequencing reveals dissociation-induced gene expression in tissue subpopulations. Nat. Methods *14*, 935–936.

Viganò, F., Möbius, W., Götz, M., and Dimou, L. (2013). Transplantation reveals regional differences in oligodendrocyte differentiation in the adult brain. Nat. Neurosci. *16*, 1370–1372.

von Schimmelmann, M., Feinberg, P.A., Sullivan, J.M., Ku, S.M., Badimon, A., Duff, M.K., Wang, Z., Lachmann, A., Dewell, S., Ma'ayan, A., et al. (2016). Polycomb repressive complex 2 (PRC2) silences genes responsible for neurodegeneration. Nat. Neurosci. *19*, 1321–1330.

Wake, H., Ortiz, F.C., Woo, D.H., Lee, P.R., Angulo, M.C., and Fields, R.D. (2015). Nonsynaptic junctions on myelinating glia promote preferential myelination of electrically active axons. Nat. Commun. *6*, 7844.

Warf, B.C., Fok-Seang, J., and Miller, R.H. (1991). Evidence for the ventral origin of oligodendrocyte precursors in the rat spinal cord. J. Neurosci. *11*, 2477–2488.

Young, K.M., Psachoulia, K., Tripathi, R.B., Dunn, S.J., Cossell, L., Attwell, D., Tohyama, K., and Richardson, W.D. (2013). Oligodendrocyte dynamics in the healthy adult CNS: evidence for myelin remodeling. Neuron *77*, 873–885.

Young, M.D., Wakefield, M.J., Smyth, G.K., and Oshlack, A. (2010). Gene ontology analysis for RNA-seq: accounting for selection bias. Genome Biol. *11*, R14.

Zeisel, A., Muñoz-Manchado, A.B., Codeluppi, S., Lönnerberg, P., La Manno, G., Juréus, A., Marques, S., Munguba, H., He, L., Betsholtz, C., et al. (2015). Brain structure. Cell types in the mouse cortex and hippocampus revealed by single-cell RNA-seq. Science *347*, 1138–1142.

Zhang, Y., Chen, K., Sloan, S.A., Bennett, M.L., Scholze, A.R., O'Keeffe, S., Phatnani, H.P., Guarnieri, P., Caneda, C., Ruderisch, N., et al. (2014). An RNA-sequencing transcriptome and splicing database of glia, neurons, and vascular cells of the cerebral cortex. J. Neurosci. *34*, 11929–11947.

Zhao, S., Guo, Y., Sheng, Q., and Shyr, Y. (2014). Advanced heat map and clustering analysis using heatmap3. BioMed. Res. Int. *2014*, 986048.

STAR★METHODS

KEY RESOURCES TABLE

REAGENT or RESOURCE	SOURCE	IDENTIFIER
Antibodies		
Chicken polyclonal anti-GFP	Abcam	ab13970, RRID:AB_300798
Goat polyclonal anti-PDGFRA	R&D	AF1062, RRID:AB_2236897
Rabbit polyclonal anti-COL1A1	Abcam	ab21286, RRID:AB_446161
Mouse monoclonal anti-CC1 (anti-APC)	Millipore	OP80, RRID:AB_2057371
Rabbit polyclonal anti-NG2	Millipore	AB5320, RRID:AB_11213678
Experimental Models: Organisms/Strains		
Pdgfrα-CreER^{T1}/RCE (mouse)	The Jackson Laboratory	B6N.Cg-Tg(Pdgfra-CreERT)467Dbe/J crossed with Gt(ROSA)26Sortm1.1(CAG-EGFP)Fsh/Mmjax
Pdgfrα-H2BGFP (mouse)	Philippe Soriano	B6.129S4-Pdgfratm11(EGFP)Sor/J
Critical Commercial Assays		
Neural tissue dissociation kit (P)	Miltenyi	130-092-628
C1™ Single-Cell Reagent Kit for mRNA Seq	Fluidigm	100-6201
miRNeasy micro kit	Qiagen	217084
MaxTract High density	Qiagen	29046
QuBit RNA HS assay kit	ThermoFisher	Q32852
RNA 6000 Pico Kit	Agilent	5067-1513
TruSeq Stranded Total RNA Library Prep Kit	Illumina	20020596
Other		
C1™ Single-Cell Open App™ IFC, 10–17 μm	Fluidigm	100-8134
C1 instrument	Fluidigm	N/A
FACSAria III Cell Sorter B5/R3/V3	BD biosciences	N/A
Axopatch 200B Amplifier	Molecular Devices	N/A
Digidata 1322A	Molecular Devices	N/A
Nikon Ti-E with motorized stage	Nikon	N/A
7900HT Fast System	Applied biosystems	N/A
Software and Algorithms		
FastQC	Andrews (2010)	https://www.bioinformatics.babraham.ac.uk/projects/fastqc/
STAR (v.2.5.0a)	Dobin et al. (2013)	https://github.com/alexdobin/STAR
GENCODE M8 annotations	Mudge and Harrow (2015)	https://www.gencodegenes.org/
featureCounts v1.5.0-p1	Liao et al. (2014)	http://bioinf.wehi.edu.au/featureCounts/
Bioconductor packages	Gentleman et al. (2004)	https://www.bioconductor.org/
biomaRt library	Durinck et al. (2009)	https://bioconductor.org/packages/release/bioc/html/biomaRt.html
limma package	Ritchie et al. (2015)	http://bioconductor.org/packages/release/bioc/html/limma.html
DESeq2 package	Love et al. (2014)	http://bioconductor.org/packages/release/bioc/html/DESeq2.html
heatmap3 library	Zhao et al. (2014)	https://cran.r-project.org/web/packages/heatmap3/index.html
RNASeqPower library	Hart et al. (2013)	https://bioconductor.org/packages/release/bioc/html/RNASeqPower.html
topGO R package	Alexa and Rahnenfuhrer (2016)	https://bioconductor.org/packages/release/bioc/html/topGO.html

(*Continued on next page*)

Continued		
REAGENT or RESOURCE	SOURCE	IDENTIFIER
Fisher-elim algorithm	Alexa et al. (2006)	https://bioconductor.org/packages/3.7/bioc/vignettes/topGO/inst/doc/topGO.pdf
Cytoscape	Cline et al. (2007)	http://www.cytoscape.org/
EnrichmentMap plugin	Merico et al. (2010)	http://baderlab.org/Software/EnrichmentMap
HISAT2 version 2.0.5	Kim et al. (2015)	https://ccb.jhu.edu/software/hisat2/index.shtml
Stringtie 1.3.1c	Pertea et al. (2015)	http://www.ccb.jhu.edu/software/stringtie/
BackSPIN2 algorithm	Marques et al. (2016)	https://github.com/linnarsson-lab/BackSPIN
Rpackage DPT	Haghverdi et al. (2016)	https://www.rdocumentation.org/packages/destiny/versions/2.0.4/topics/DPT
PAGODA (SCDE R-package)	Fan et al. (2016)	http://hms-dbmi.github.io/scde/
MAST package R	Finak et al. (2015)	https://github.com/RGLab/MAST
SCN3E	This paper	https://github.com/Castelo-Branco-lab/OPCsinglecell2017
Deposited Data		
Raw and Analysed data	This paper	GEO: GSE95194 (single cell) and GEO: GSE95093 (bulk)
Other		
Webresource with browsable data	This paper	https://ki.se/en/mbb/oligointernode

CONTACT FOR REAGENT AND RESOURCE SHARING

Further information and requests for resources and reagents should be directed to and will be fulfilled by the Lead Contact, Gonçalo Castelo-Branco (goncalo.castelo-branco@ki.se).

EXPERIMENTAL MODEL AND SUBJECT DETAILS

Mice

Mice line used in this study included Pdgfra-cre-ERT/RCE (mixed C57BL/6NJ and CD1 background), which is result of a cross between the Pdgfra-cre-ERT line (Kang et al., 2010) and the Z/EG line (Butt et al., 2008) and the Pdgfra-H2BGFP knock-in mouse (Klinghoffer et al., 2002) (background C57BL/6NJ). Female Pdgfra-cre-ERT/RCE and the resulting embryos (E12.5) or pups (P3) were used for lineage tracing. No further behavioral experiments were done in this group of animals.

Homozygous Pdgfra-H2BGFP, in which H2B-eGFP fusion gene is expressed under the promoter of the OPC marker, *Pdgfra* have an embryonic lethal phenotype, with half of the embryos failing to survive past embryonic day 12.5 and the remainder failing to survive beyond embryonic day 15.5 (https://www.jax.org/strain/007669). Therefore, we used heterozygote mice, in which *Pdgfra* is expressed mainly in OPCs but the GFP remains to some extent in the early stages of OL differentiation, due to GFP half-life (Clarke et al., 2012).

Mice were time mated to obtain embryos with 13.5 days or post-natal day 7 pups. Gender was randomized since the experiments were mainly done in embryos and pups. The following light/dark cycle was used: dawn 6.00-7.00; daylight 07.00-18.00; dusk 18-00-19.00; night 19.00-06.00. A maximum of 5 adult mice per IVC-cage of type II Allentown. Breedings were done with 1 male and up to 2 females. All experimental procedures performed followed the guidelines and recommendations of local animal protection legislation and were approved by the local committee for ethical experiments on laboratory animals (Stockholms Norra Djurförsöksetiska Nämnd in Sweden).

METHOD DETAILS

OPC Extraction

Embryos with 13.5, E17.5 days and pups from post-natal day 7, from both genders of the Pdgfra-H2BGFP mice line were used to extract OPCs. The dissociation method varied depending on the stage. For embryonic stage, forebrain and spinal cord were excised and tissue was mechanical dissociated in HBSS with ions using 3 Pasteur pipettes of decreasing diameter. For post-natal stages, the same tissues were dissociated with a Papain Neural dissociation kit from Miltenyi, following the manufacturer's instructions.

FACS Sorting

The single-cell suspension from embryonic and post-natal tissue was FACS sorted for GFP cells using a BD FACSAria III Cell Sorter B5/R3/V3 system. For bulk sequencing, cells were collected in non-sticky RNAse free tubes containing RNA Later, Qiazol was added

and samples were snap frozen until further RNA extraction. For this, pooling of mice samples was performed to obtain at least 50,000 cells. For single-cell analysis, cells were collected in cutting solution with 1% BSA and quickly prepared for capture on the C1 fluidigm system. FACS gating strategy is presented in Figure S1. We observed a gradient of GFP+ cells at P7. This gradient was also previously observed in other studies with the Pdgfra-H2BGFP mice, where the OPC population was selected for the expression of high levels of GFP (Moyon et al., 2015). We performed qRT-PCR studies and determined that while the GFP++ population expressed *Pdgfra*, *Olig2* and *Plp1*, the GFP+ population lacked expression of these markers (data not shown, n = 1). Since the main focus of our manuscript is OPCs, which express *Pdgfra*, we did not proceed to the analysis of the GFP+/Pdgfra- population.

RNA Extraction for Bulk RNA-Seq

RNA was extracted with miRNeasy micro kit from Qiagen, following manufacturer's instructions with minor modifications. Briefly, Qiazol extracts were thawed and vortexed for 1min. Chloroform was applied and mixture was transferred to a MaxTract High density column. From that point on, the extraction was then performed according to the kit's manual for low input samples. RNA was then measured with QuBit RNA HS assay kit and Agilent RNA 6000 Pico Kit. 50 ng of RNA from each E13.5/ P7 sample was used for library preparation. Each sample represented a pool of *Pdgfra*+ cells collected from 4 – 32 animals to achieve the 50ng required for library preparation.

Library Preparation for Bulk RNA-Seq

Illumina's TruSeq Stranded Total RNA Library Prep Kit was used according to manufacturer's instructions. Three pooled biological replicates were sequenced for each developmental stage and CNS region, and a total of 38 - 66 million 150 bp strand-specific paired-end reads were generated for each replicate, comprising a total of 620.5 million reads across all datasets (Figure S1).

Single-Cell RNA-Seq Cell Capture and Imaging

Cell suspensions in a concentration of 600-1000 cells/μL was used. C1 Suspension Reagent was added (all 'C1' reagents were from Fluidigm, Inc.) in a ratio of 4μL to every 7μL cell suspension. 11μL of the cell suspension mix was loaded on a C1 Single-Cell AutoPrep IFC microfluidic chip designed for 10- to 17μm cells, and the chip was then processed on a Fluidigm C1 instrument using the 'mRNA Seq: Cell Load (1772x/1773x)' script (30 min at 4°C). The plate was then transferred to an automated microscope (Nikon TE2000E), and a bright-field image (20× magnification) was acquired for each capture site using μManager (http://micro-manager.org/ (2)), which took <15 minutes. Quality of cells, control for doublets and processing of C1 chips were performed in the Eukaryotic Single Cell Genomics facility at SciLife Lab, as described in Zeisel et al. (2015) and Marques et al. (2016).

Lineage Tracing

Pdgfra-cre-ERT/RCE female mice were injected 2mg tamoxifen (20mg/ml, Sigma) at pregnancy day E.12.5 (txE12.5) or when pups were P3 (txP3). Brain tissue was then harvested at P7-P8 for electrophysiology and single-cell RNA sequencing and at P21 for immunohistochemistry experiments, respectively. Since the Pdgfra-cre-ERT line was crossed with a low efficiency Z/EG reporter and not a R26-GFP reporter, the likelihood of sporadic tamoxifen-independent recombination of the Pdgfra-CreERT mouse line in this study is low (http://jackson.jax.org/rs/444-BUH-304/images/18280_Bergles.pdf).

Electrophysiology

P7-P8 pups were deeply anesthetized with isoflurane and brains were collected in ice-cold solution of the following composition (in mM): 62.5 NaCl, 100 sucrose, 2.5 KCl, 25 $NaHCO_3$, 1.25 NaH_2PO_4, 7 $MgCl_2$, 1 $CaCl_2$, and 10 glucose. Brains were vibratome sectioned to 300μm slices in the same solution and were then let recover for 1 h at room temperature in oxygenated aCSF (in mM): 125 NaCl, 2.5 KCl, 25 $NaHCO_3$, 1.25 NaH_2PO_4, 1 $MgCl_2$, 1 $CaCl_2$, and 10 glucose. To maximize the selection of OPCs for electrophysiological recordings, we targeted small circular eGFP+ cells found in the corpus callosum, especially avoiding elongated cells attached to blood vessels (which would be VLMCs or pericyte-lineage cells, labeled in this mouse line).

Whole-cell patch-clamp recordings were performed at 25±2°C, with slices continuously perfused with oxygenated aCSF. Patch electrodes were made from borosilicate glass (resistance 5–8 MΩ; Hilgenberg, GmbH) and filled with a solution containing (in mM): 130 CsCl, 4 NaCl, 0.5 $CaCl_2$, 10 HEPES, 10 EGTA, 4 MgATP, 0.5 Na_2GTP. Neurobiotin (0.5%, Vectorlabs) was included for post-hoc identification of recorded cells.

Cells were recorded in voltage-clamp mode held at -70 mV. At the end of the recording tetrodoxin (TTX, 10μM) or CNQX/MK-801 (10μM /5μM) were applied for 10 min to block spontaneous neuronal activity or glutamatergic inputs respectively. Currents were recorded with an Axopatch 200B amplifier (Molecular Devices), sampled at 10 kHz and digitized with Digidata 1322A (Molecular Devices). All drugs were ordered from Sigma. In order to confirm their OPC identity, slices were fixed with 4% PFA for 1-2 h after recording, washed and kept in PBS 4°C until stained for NG2 (rabbit anti-NG2 1:200, Millipore), Streptavidin 555 (1:1000, Invitrogen) and Alexa-647 anti-rabbit (1:400, ThermoFisher). We did not recover post-staining for every recorded cell included in the analysis. Nevertheless, we enriched our sample for OPCs, using a reporter mouse line and targeting small round eGFP+ cells. Accordingly, all cells have homogenous intrinsic properties (e.g., input resistance – which differs greatly in more mature stages). Furthermore, in relation to synaptic inputs we recovered eGFP+/NG2+ cells comprising both the lowest and the highest frequencies of synaptic events.

7 slices from 2 txE13.5 animals and 15 slices from 4 txP3 animals were recorded. In electrophysiology it is common to recorder from 6-10 cells per group. After a first batch of experiments, we appreciated that the txP3 group was more heterogeneous (number of events) and we decided to enlarge the sample to 15 cells in order to confirm the synaptic events distribution.

Electrophysiology Analysis

All traces were low-pass filtered at 1KHz (8-pole Bessel filter) and only events with amplitude larger than -10pA were included in the analysis. We utilized a semi-automated event-detection on Clampex with which events were visually inspected and unclear cases were discarded. Cells that died during recording or whose signals were not analyzed were excluded.

Immunohistochemistry

Pdgfra-CreERT/RCE mice recombined with Tamoxifen at E12.5 were perfused at P20 with PBS followed by 4% PFA. Brains and spinal cords were dissected and postfixed with 4% PFA for 2h, at 4°C. The tissues were then cryoprotected with a 30% sucrose solution for 48 hours. The tissues were embedded into OCT (Tissue-Tek) and sectioned coronally (20 um thickness).

Sections were quickly boiled in antigen retrieval (Dako, S1699) and stood in the antigen solution until cooling down. They were then permeabilized in PBS/0.3% Triton (Millipore) 3× 5 minutes and blocked for 1 hour in PBS/0.3% Triton/5% normal donkey serum (Sigma, D9663) at room temperature. Sections were incubated overnight at 4°C with primary antibodies [GFP (Abcam, ab13970, chicken 1:2000), PDGFRA (R&D, AF1062, Goat 1:200), COL1A1 (Abcam, ab21286, rabbit, 1:50) or CC1 (anti-APC; Millipore, OP80, Mouse 1:100) diluted in PBS/0.3% Triton/2% normal donkey serum. After washing the sections 3x 5min with PBS, secondary Alexa Fluor-conjugated antibodies (Invitrogen, Alexa Fluor 488 1:500, Alexa Fluor 555 1:1000 and Alexa Fluor 647 1:250) diluted in PBS/0.3% Triton/2% normal donkey serum were added and incubated for 1 hour at room temperature. Thereafter, slides were mounted with mounting medium containing DAPI (Vector, H-1200) and kept at 4°C until further microscopic analysis.

Antibodies have been cited by other authors, are available on the webpage of the provider company or have been tested for immunohistochemistry in mouse by the company.

Microscopy

Combined images of DAPI, Alexa 555, Alexa 488 and Alexa 647, spanning the corpus callosum (CC) and dorsal horn were obtained in a Zeiss LSM700 Confocal. For quantifications, 3 animals were used in each timepoint and 4-5 slices were photographed per animal. An average of 33 and 46 photos in CC and dorsal horn respectively were counted per animal. The number of animals used was similar to those reported in previous publications presenting similar experiments (Kessaris et al., 2006). All the countings were normalized to the area analysed in each photo. % of Pdgfra+Col1a1- (OPCs) and Pdgfra+Col1a1+ (VLMCs) cells out of GFP were calculated. The remaining GFP cells (Pdgfra-/Col1a1-) were considered as non-OPC cells. The percentage of GFP cells out of the Pdgfra+ population was counted as being OPCs derived from the E13.5 wave while the remaining Pdgfra+GFP- were considered to derive from the second/third waves.

QUANTIFICATION AND STATISTICAL ANALYSIS

Statistical Analysis of Gene Expression of Bulk RNA-Seq

After read quality control using FastQC (Andrews, 2010), STAR (Dobin et al., 2013) (v.2.5.0a) was used to map reads to the mouse mm10 genome. GENCODE (Mudge and Harrow, 2015) M8 annotations were used to construct the splice junction database and as a reference for the count tables. Gene-level count tables were obtained using featureCounts (Liao et al., 2014) v1.5.0-p1, assigning multi-mapping reads fractionally to their corresponding loci and using convergent rounding to convert the resulting count tables to the nearest integer values. Bioconductor packages were used for data processing and analysis (Gentleman et al., 2004), and the biomaRt library was used for querying annotations and mapping across gene identifiers (Durinck et al., 2009). The limma package was used for differential gene expression analysis (Ritchie et al., 2015), with normalisation carried out using the voom approach (Law et al., 2014). Genes were tested for differential expression if they displayed 0.7 counts per million in at least three of the libraries, and considered differentially expressed if found to have a Benjamini-Hochberg adjusted p-value < 0.05 and a greater than twofold change in expression between queried samples. Exploratory data analysis and principle component analysis visualisation was carried out using the DESeq2 package (Love et al., 2014). Heatmaps were generated using the heatmap3 library (Zhao et al., 2014), using the Spearman correlation coefficient between counts per million per gene as the distance metric for clustering.

3 replicates for each time point/tissue was run for RNA seq. Recent guidelines for "A survey of best practices for RNA-seq data analysis" indicates that "three replicates are the minimum required for inferential analysis" (Conesa et al., 2016). We used the RNASeqPower library (Hart et al., 2013) to perform power calculations on the count tables obtained after filtering out lowly expressed genes. The lowest median depth was observed in the E13.5S3 dataset, with a median number of 90 reads per tested gene in the library. Using this value as the coverage parameter for RNASeqPower, we revealed that with a within-group biological coefficient of variation of 0.1, which is commonly used for inbred animals and with the 0.05 size of the test statistic we used (alpha) and a power of 0.95, we would have needed 1.14 samples per group to accurately quantitate differential gene expression with a logFC > 2. Hence, our analysis was adequately powered.

All tests used are in accordance with current best practices as outlined by Conesa et al. (2016) and analysis are carried out in line with current best practices. All quality control metrics (% mapping, reads to genes etc) were consistent between the replicate samples.

Gene Ontology

Gene ontology analysis was carried out using the topGO R package (Alexa and Rahnenfuhrer, 2016) using the Fisher-elim algorithm (Alexa et al., 2006). The Ensembl version 83 annotation was downloaded from biomaRt and used to generate the gene:category mappings. To take into account length bias in RNA-Seq gene ontology enrichment analysis (Young et al., 2010) a list of genes expressed at similar levels but not differentially expressed between the two conditions was used, selecting 10 non-differentially expressed genes for every differentially expressed one. The union of these genes for each condition was used as the background list for topGO. For visualization, the results of the topGO analysis, expression values and the gene ontology mappings were exported to Cytoscape (Cline et al., 2007), and the EnrichmentMap plugin (Merico et al., 2010) was used for visualizing the enriched categories and the overlap between them. The size of each circle in Figures 1E and 1F indicates the number of genes contributing to each gene ontology category, while the thickness of the connections between circles indicates the degree of overlap between the two categories presented.

Single-Cell RNA-Seq

List of All the Single-Cell Experiments

2 E13.5 Brain experiments: 2 and 4 Fluidigm chips, respectively
1 E13.5 Spinal cord experiment – 4 Fluidigm chips
1 P7 Brain experiment – 4 Fluidigm chips
2 P7 Spinal cord experiments – 1 and 4 Fluidigm chips, respectively
2 experiments with P7 /P8 brain from E13.5 derived cells – 6 Fluidigm chips
1 experiment with P7/P8 brain from P3 derived cells – 3 Fluidigm chips
1 experiment with E17.5 brain – 1 Fluidigm chip

To prevent batch effects, in a balanced study design cells from different timepoints and regions would be mixed in each processed single-cell run. Since we were collecting several timepoints and regions and using the Fluidigm C1 96 well Chips, this was not possible to implement. Cells from the same area and age were collected and processed at several time points. We did not observe differences between the experiments that would dramatically affect the analysis of the data, as assessed by comparison of the original dataset with our dataset where the confounding factors were regressed out.

Single-Cell Clustering

Quality Control

Prior to clustering, cells from our dataset and the OPC, COP, and VLMC cells from Marques. et. al (Marques et al., 2016) were selected based on a minimum transcript threshold. Cells had to express a minimum of 1000 mRNA molecules per cell excluding mitochondrial RNA (filtered by the string "Mt-"), and repeat RNA (filtered by the string "r_") in order to be considered for analysis. Distributions of transcript counts and total gene counts were calculated and categorized by cluster (Figure S1). Additionally 84 cells were considered doublets due to a joint expression of either OL and neuronal genes, or a joint expression of OPC and VLMC associated genes.

Weak single-cell data / dead cells which did not pass the quality control check have been filtered away from the single-cell analysis.

BackSPIN Clustering

The post QC dataset was clustered using the BackSPIN2 algorithm as previously described (Marques et al., 2016; Romanov et al., 2017). In short, the algorithm is an adaptation to the sorting algorithm SPIN (Tsafrir et al., 2005) wherein a bi-clustering is performed by sorting the cells and genes into a one-dimensional ordering where a binary split is performed based on the distribution of genes within each ordering. The algorithm repeatedly performs feature selection and subsequent splits until a certain threshold is achieved.

Pathway and Geneset Overdispersion Analysis (PAGODA)

The post QC dataset was clustered using PAGODA (SCDE R-package) (Fan et al., 2016). First, the drop-out rate is determined and the amplification noise is estimated through the use of a mixture-model. Then the first principal component is calculated for all gene sets and GO-term clusters are provided or identified, and over-dispersion is defined as the amount of variance explained by the gene set above expectation. Holm procedure is used as a part of the SCDE package.

Unbiased Cluster Determination PAGODA

Cluster generation within PAGODA is based on hierarchical clustering of the distance measure obtained from the first principal component of the gene sets. We developed an algorithm to determine the final cluster amount in an unbiased way. First, we perform a differential expression analysis using SDCE for each new split to calculate for each cluster what genes are differentially expressed between them, we filter genes by a requirement to be expressed in at least 60% of the population, and then we calculate a p-value assuming a normal distribution based on the Z-scores obtained from the differential expression analysis. Subsequently we either discard the split or accept the split based on the significance of the top 20 most significant genes ($p<0.01$). Using these settings the algorithm returned 15 clusters from the dataset.

Merging of BackSPIN2 and PAGODA Clusters

BackSPIN2 sorts the expression matrix by genes and cells, then splits the cells iteratively forming clusters with associated genes. Although bi-clustering (both cells and genes) is an advantage, genesets that are not unique to only one cluster can be discarded, reducing sub-clustering power. PAGODA models the expression matrix, filtering out possible dropout genes. The residual expression data is then assigned to pathways and genesets, which show coordinated variability in the dataset, and used to hierarchically cluster the data in a set number of clusters. PAGODA is robust to noise through the use of co-varying genesets, potentially detecting variations in gene expression that would be insignificant when measured using any one single gene. Nevertheless, PAGODA's Bayesian modelling is slow and the number of clusters has to be set manually. In order to establish an unbiased end-point for cluster splitting and avoid oversplitting in PAGODA, we implemented a custom algorithmic threshold, which performs differential gene expression between clusters, and indicates whether they should be merged if no statistical difference in gene expression is detected. In general, PAGODA and BackSPIN2 gave rise to clusters with similar transcriptional profiles. While we adopted the main BackSPIN2 clusters, PAGODA resolved OPCs as three populations and not as one population (as in BackSPIN2). This might be due to low expression and the possible quiescent phenotype of some of the OPC clusters. PAGODA has the advantage of looking at patterns of groups of genes instead of individual genes thus increasing sensitivity for low expressing cells. Therefore we retained the three found OPC clusters in PAGODA and the NP3 cluster from PAGODA (due to their low expression) and merged them with the remaining BackSPIN2 clusters. The remaining cells that were classified by backSPIN2 as OPCs but not by PAGODA, were reassigned to the COP and NP3 clusters, given their transcriptional profile.

BackSPIN2 clustering indicated distinct clusters within the NFOL cluster. Enrichment analysis and marker selection revealed shared markers and enriched genes as well as some minor differences in genes such as *Mog* and *Mag* expression. Further analysis of these subclusters did not fall within the scope of this paper and as such the clusters were merged into the NFOL cluster. Furthermore, Enrichment analysis and marker selection of the NP1 cluster revealed a clear bimodal expression profile, hence we used the BackSPIN2 clustering data to split one level deeper within the NP1 population resulting in the NP1a and NP1b subclusters. PAGODA also revealed these when allowed to oversplit, our splitting algorithm indicated this split to form valid clusters. However, the NP1 cluster split was preceded by a number of non-valid splits, meaning that this cluster is a subcluster and statistical evaluation of split validity is less effective due to the small cluster size.

Single-Cell Near-Neighbor Network Embedding (SCN3E)

Feature Selection

Initial gene selection involves a cutoff for expressed genes determined by expression above the mean expression of all genes, then a feature selection is performed using the coefficient of variation, and for each gene we select genes above the support vector regression fitted line.

Dimensional Reduction of Count Based Expression Matrix Spaces

For dimensional reduction we use diffusion mapping, a non-linear dimensional reduction technique (Rpackage DPT) (Haghverdi et al., 2016) including a locally scaled transition matrix for improved resolution. We estimate the number of diffusion components using the elbow method and overcluster the data in 100 clusters. Diffusionpseudotimes are then calculated for each of the 100 clusters of which the first 20 principle components are used for subsequent network embedding (depending on the complexity of the dataset.

Nearest Neighbor Network Embedding and Lineage Estimation

The matrix of pseudotimes with origin of each previously determined cluster are reduced using PCA. Distances between cells are defined using Manhattan distances. Per individual cell, the 20 nearest neighbors are calculated (within certain parameters, see accompanying code) and network edges are created between them. Edges are then weighted based on the Pearson correlation. The edge weights are then raised to a power (soft-thresholding). Generating a network based on transition probabilities (pseudotimes) will emphasize smooth transitions and break up spurious connections in the graph.

Differential Gene Expression

Differential gene expression for scRNA-seq data was performed using MAST (Finak et al., 2015), and the SCDE R-package (Fan et al., 2016). All enrichments were calculated using the geneselection obtained from the previously mentioned support vector model fitted variable geneselection method. All differential expression analyses were performed using default settings.

Electrophysiology Analysis

For each cell, average amplitude and frequency values were calculated by total number events per time. Data are mean ± s.e.m. P-values are from Student's two independent sample ($p = 0.27$; frequency $F = 13.95$, $t = -1.53$, $df = 20$; amplitude ($F = 0.99$, $t = 1.1$, $df = 18$)) or paired t-tests ($p = 0.033$, $p=0.06$ and $p=0.11$; paired txE12.5 x txE12.5+TTX ($t=2.497$, $df = 4$); paired txP3 x txP3+TTX ($t = 2.4974$, dff = 2); paired txP3 x txP3+CM ($t = 3.71$, dff = 3)). *, p-value < 0.05.

Microscopy Analysis

Percentages of GFP^{+}-Pdgfrα^{+} (fate mapped OPCs), GFP^{+}-Col1α1^{+} (fate mapped VLMCs), GFP^{+}-Pdgfrα/Col1α1^{-} (fate mapped oligodendrocytes) cells normalized by area were calculated. The results are expressed as means ± SEM. Statistics comparing the percentage of First and Second/third wave- derived OPCs were performed using two-tailed unpaired t test (Prism, GraphPad). Statistics comparing the percentage of the VLMCs, OPCs, and other cell populations were performed using One-way

ANOVA with Tukey's multiple comparisons test (Prism, GraphPad). *, p-value < 0.05; **, p-value < 0.01; ***, p-value < 0.001 and ****, p-value < 0.0001.

DATA AND SOFTWARE AVAILABILITY

Data Resources

The accession number for the single-cell RNA-Seq raw data reported in this paper is GEO: GSE95194 and the accession number for the bulk RNA-Seq raw data reported in this paper is GEO: GSE95093.

Software

Code used for bulk and single-cell RNA-Seq analysis is available at https://github.com/Castelo-Branco-lab/OPCsinglecell2017

ADDITIONAL RESOURCES

A web resource for browsing differential gene expression data for the single-cell and bulk data can be accessed at our resource webpage https://ki.se/en/mbb/oligointernode.

Developmental Cell, Volume *46*

Supplemental Information

Transcriptional Convergence of Oligodendrocyte Lineage Progenitors during Development

Sueli Marques, David van Bruggen, Darya Pavlovna Vanichkina, Elisa Mariagrazia Floriddia, Hermany Munguba, Leif Väremo, Stefania Giacomello, Ana Mendanha Falcão, Mandy Meijer, Åsa Kristina Björklund, Jens Hjerling-Leffler, Ryan James Taft, and Gonçalo Castelo-Branco

a

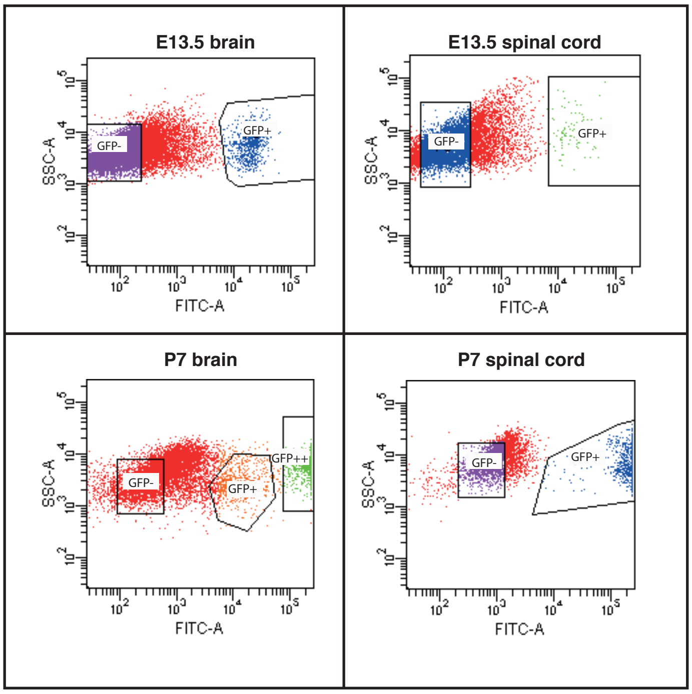

b

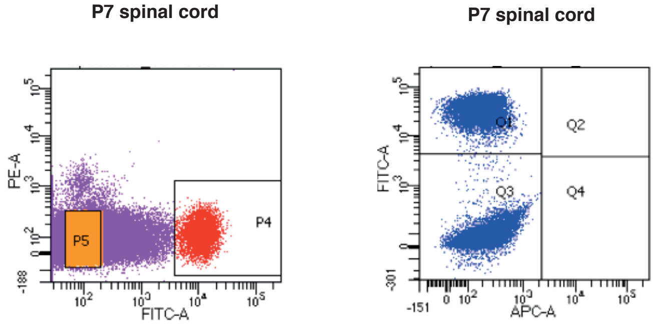

c

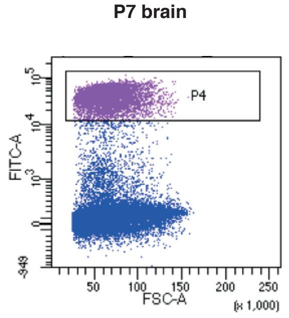

d

Dataset	Reads	Uniquely mapped	Median TIN	Spliced Reads	Stranding	Partially novel SJ	Novel SJ	Total SJ	GTAG	GCAG	ATAC	Non-canonical SJ
**E13.5B1**	46837623	87.73%	76.78	24.03%	98.87%	16%	8%	13424516	98.99%	0.86%	0.10%	0.06%
**E13.5B2**	53041658	86.42%	77.01	22.33%	98.08%	15%	8%	13995601	98.98%	0.86%	0.10%	0.06%
**E13.5B3**	42836948	83.84%	74.90	23.78%	98.29%	14%	8%	12104537	98.97%	0.87%	0.10%	0.07%
**E13.5S1**	46481407	88.30%	75.49	17.43%	98.77%	14%	8%	9710372	99.05%	0.80%	0.09%	0.06%
**E13.5S2**	60703882	89.39%	76.32	17.59%	97.63%	15%	9%	12791166	99.05%	0.80%	0.09%	0.06%
**E13.5S3**	37869824	72.44%	67.54	8.54%	98.31%	8%	4%	3883562	99.00%	0.84%	0.09%	0.06%
**P7B1**	49518204	85.75%	83.49	35.33%	67.19%	18%	9%	20712958	98.98%	0.85%	0.11%	0.06%
**P7B2**	46121858	83.53%	83.21	34.65%	60.02%	17%	9%	18910438	98.99%	0.84%	0.11%	0.06%
**P7B3**	58598593	85.69%	82.87	34.24%	81.27%	19%	10%	23710326	98.99%	0.84%	0.11%	0.06%
**P7S1**	66036696	84.30%	83.84	38.28%	89.37%	21%	10%	29950121	99.01%	0.83%	0.10%	0.06%
**P7S2**	56877838	84.34%	83.30	37.91%	79.41%	20%	9%	25495488	99.01%	0.83%	0.11%	0.06%
**P7S3**	55570228	85.17%	82.73	38.94%	76.44%	18%	9%	25793768	98.99%	0.84%	0.11%	0.07%

e

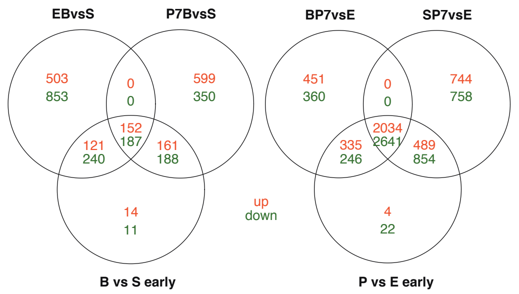

f

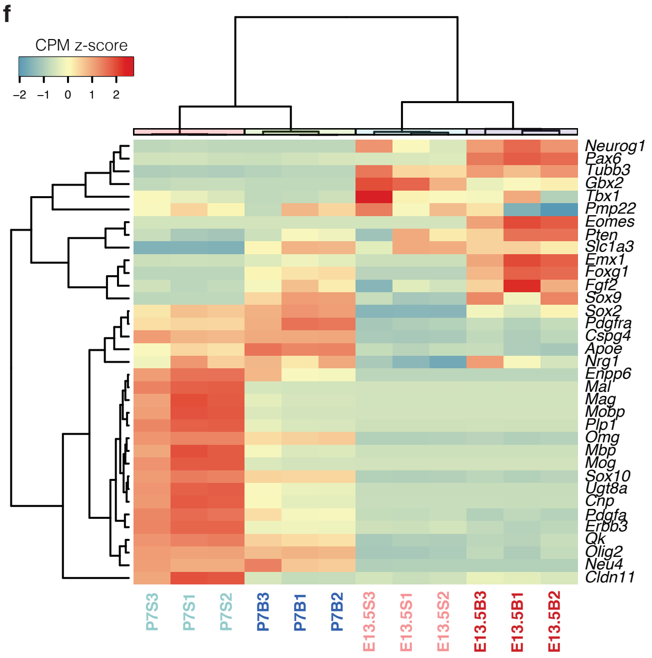

g

	Pre QC	Post QC
Pdgfra+/GFP	2496	1514
Marques et al 2016	526	460

Clusters	# cells
OPC1a	446
OPC1b	264
OPC2	86
COP	193
NFOL	144
Cycling	18
NP1a	82
NP1b	14
NP2	150
NP3	121
eVLMC	214
PLC	24
pnVLMC	148
VLMC	52

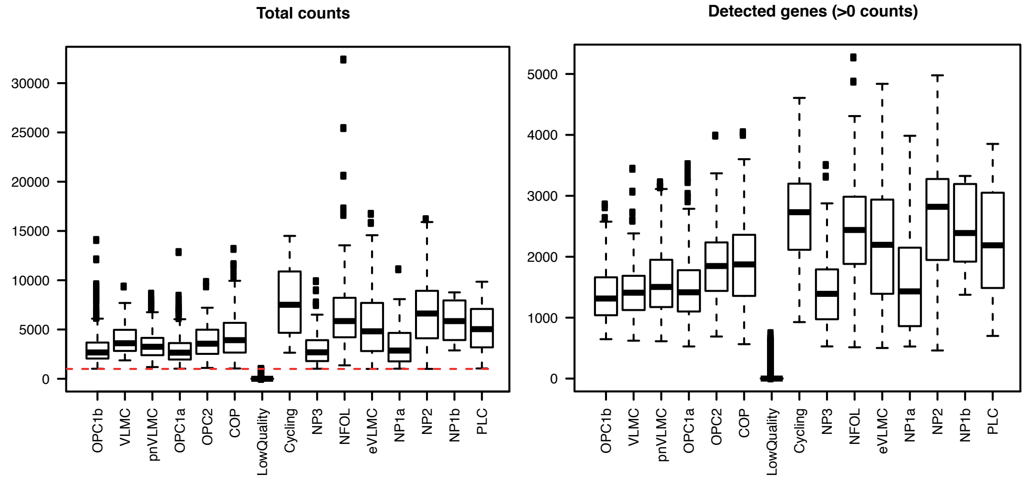

Figure S1 (related to Figures 1-2)

a) FACS sorting graph, highlighting selected Pdgfra+/GFP cells for bulk RNA-Seq;

b) FACS gating of GFP+ cells (FITC) from the P7 spinal cord of Pdgfra-H2B-GFP mice in 2 independent single cell RNA-Seq experiments.

c) FACS gating of GFP+ cells (FITC) from the P7 brain of Pdgfra-H2B-GFP mice in a single cell RNA-Seq experiment.

d) Bulk sequencing library depth and mapping statistics

e) Overlap between differentially expressed genes at each of the stages and regions investigated in the bulk RNA-Seq;

f) Heatmap showing hierarchical clustering of bulk samples based on normalised gene expression (counts per million mapped reads, cpmm) of selected genes involved in oligodendrocyte lineage progression;

g) Single-Cell RNA sequencing Quality Control.

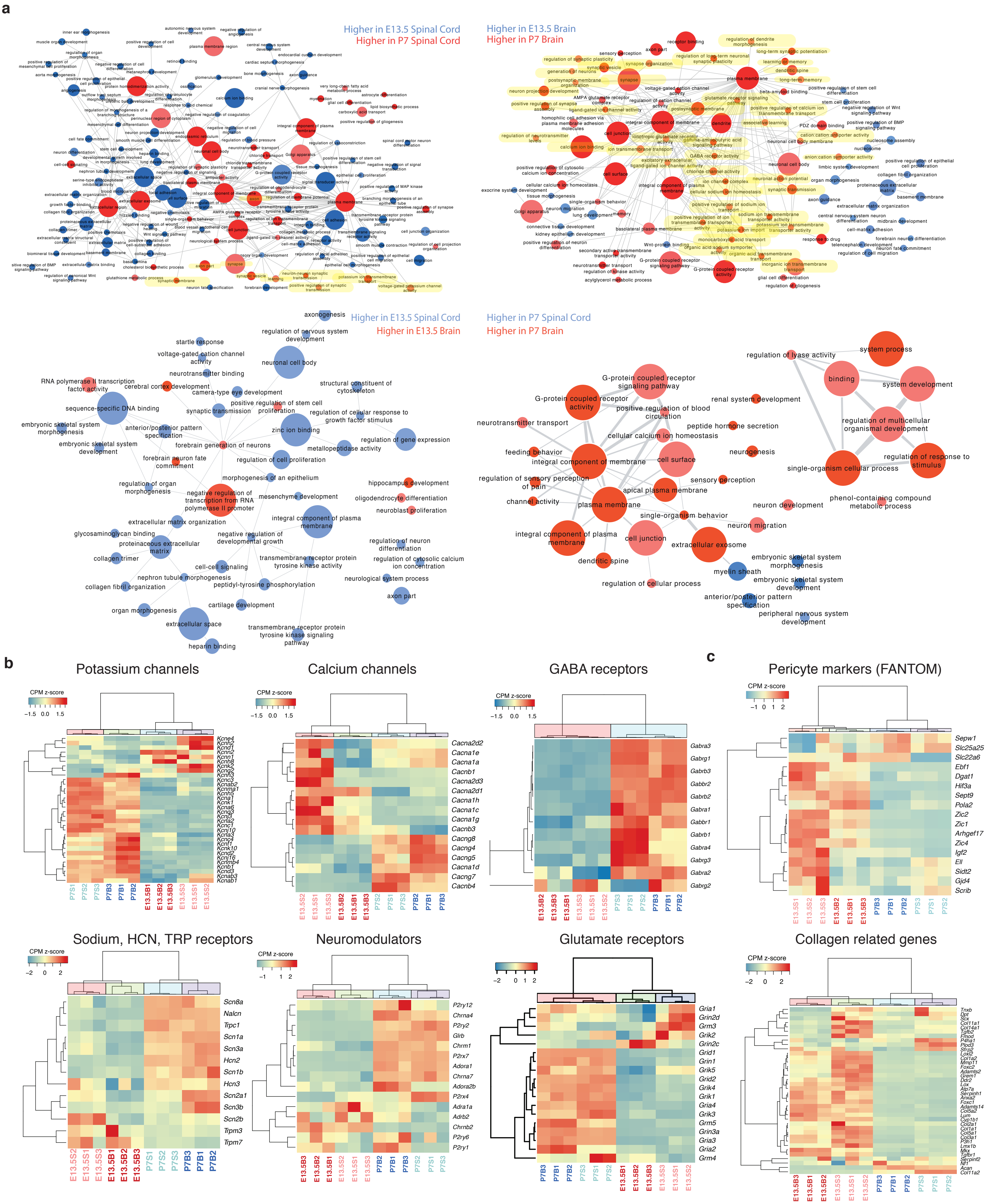

Figure S2 (related to Figure1)

a) Gene ontology analysis with enriched biological functions overrepresented in either E13.5 versus P7 brain, and E13.5 versus P7 spinal cord.

b) Hierarchical clustering of bulk samples based on normalised gene expression (cpmm) of potassium and calcium channels, GABA, Sodium, HCN and TRP receptors and neuromodulator-related, glutamate receptor genes observed in the bulk sequencing in Pdgfra+/GFP cells;

c) Hierarchical clustering of bulk samples based on normalised gene expression (cpmm) of pericyte marker and collagen related genes observed in the bulk sequencing of Pdgfra+/GFP cells

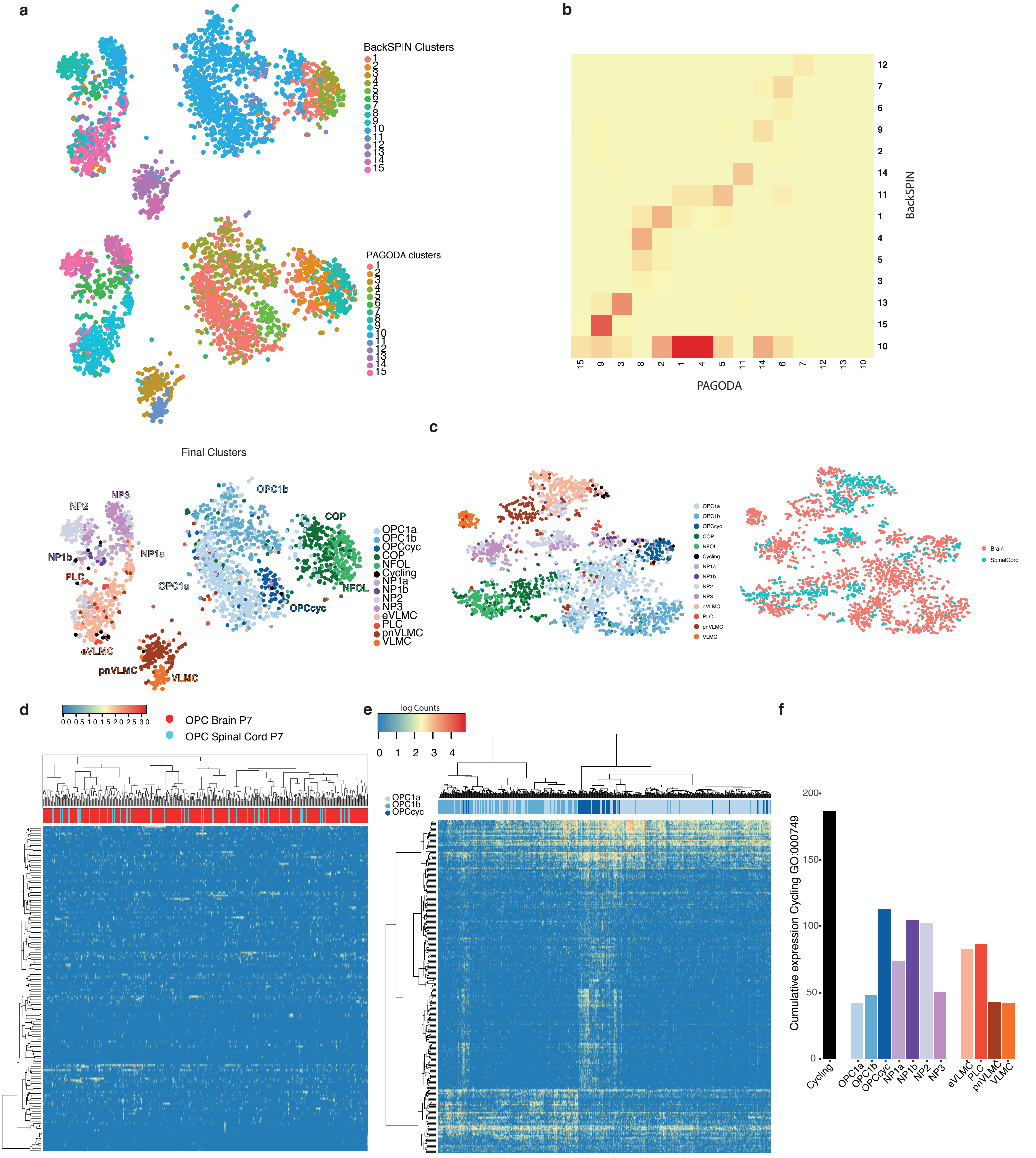

Figure S3 (related with Figure 2)

a) Differences and commonalities in the BackSPIN2 and PAGODA derived clusters, and the merged and final clusters as shown by tSNE;
b) Cluster overlap comparing PAGODA and BackSpin2, colorshaded by number of cells;
c) Noise corrected t-SNE after removal of hidden confounding factors using the f-scLVM package
d) Hierarchically clustered heatmap of top differentially expressed genes between Brain and Spinal Cord P7 OPCs, as assessed by SCDE
e) Hierarchically clustered heatmap of top differentially expressed genes between OPC1a, OPC1b, and OPCcyc, as assessed by SCDE
f) Total molecule count of cell cycle gene expression taken from GO:000749 among the different cell populations;

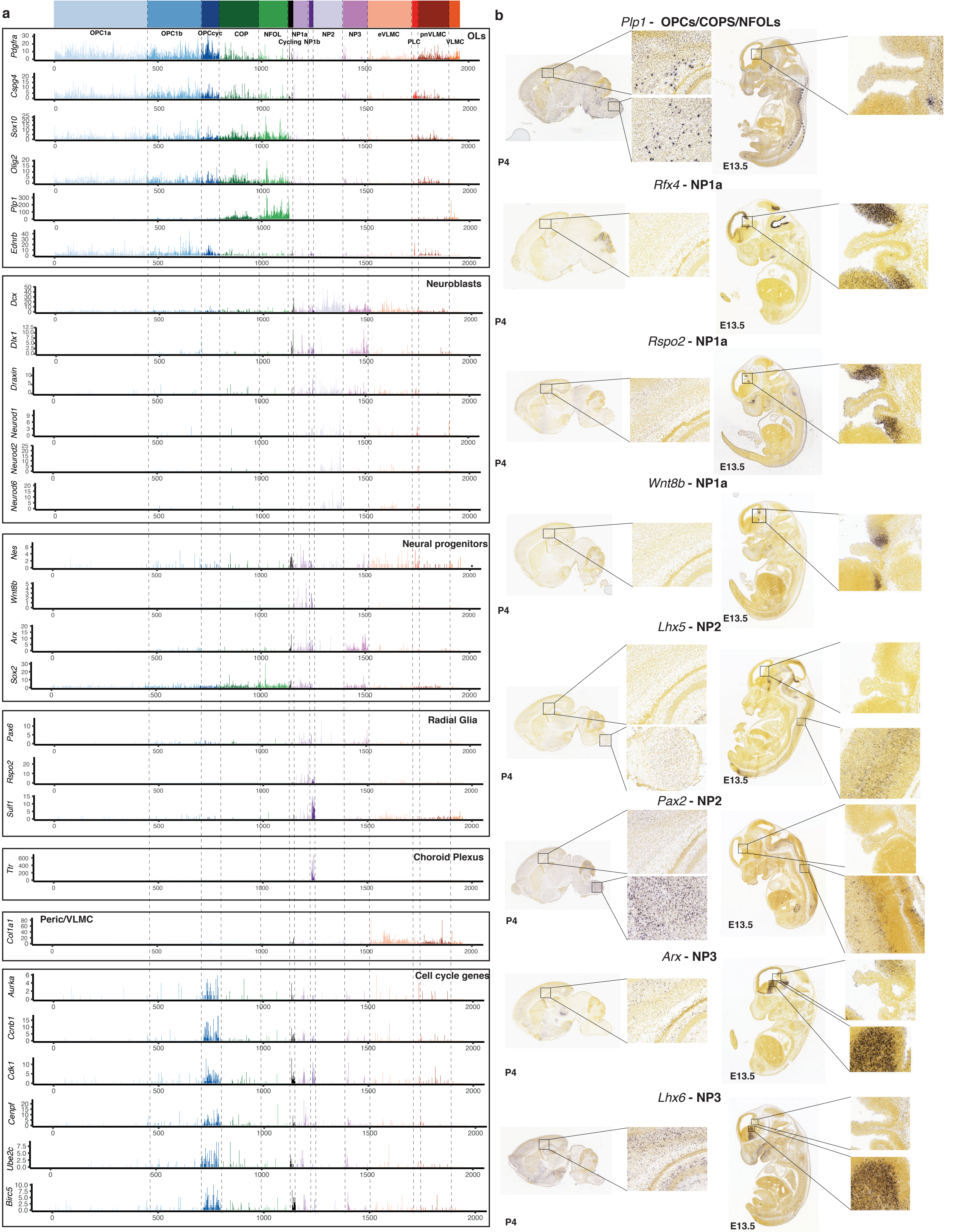

Figure S4 (related to Figure 2) a) Single cell expression of known markers of different lineages in the different clusters, and of cell cycle genes.
b) In situ hybridisation of NP markers at E13.5 and P4 mouse CNS, from @2013 Allen Developing Mouse Brain Atlas

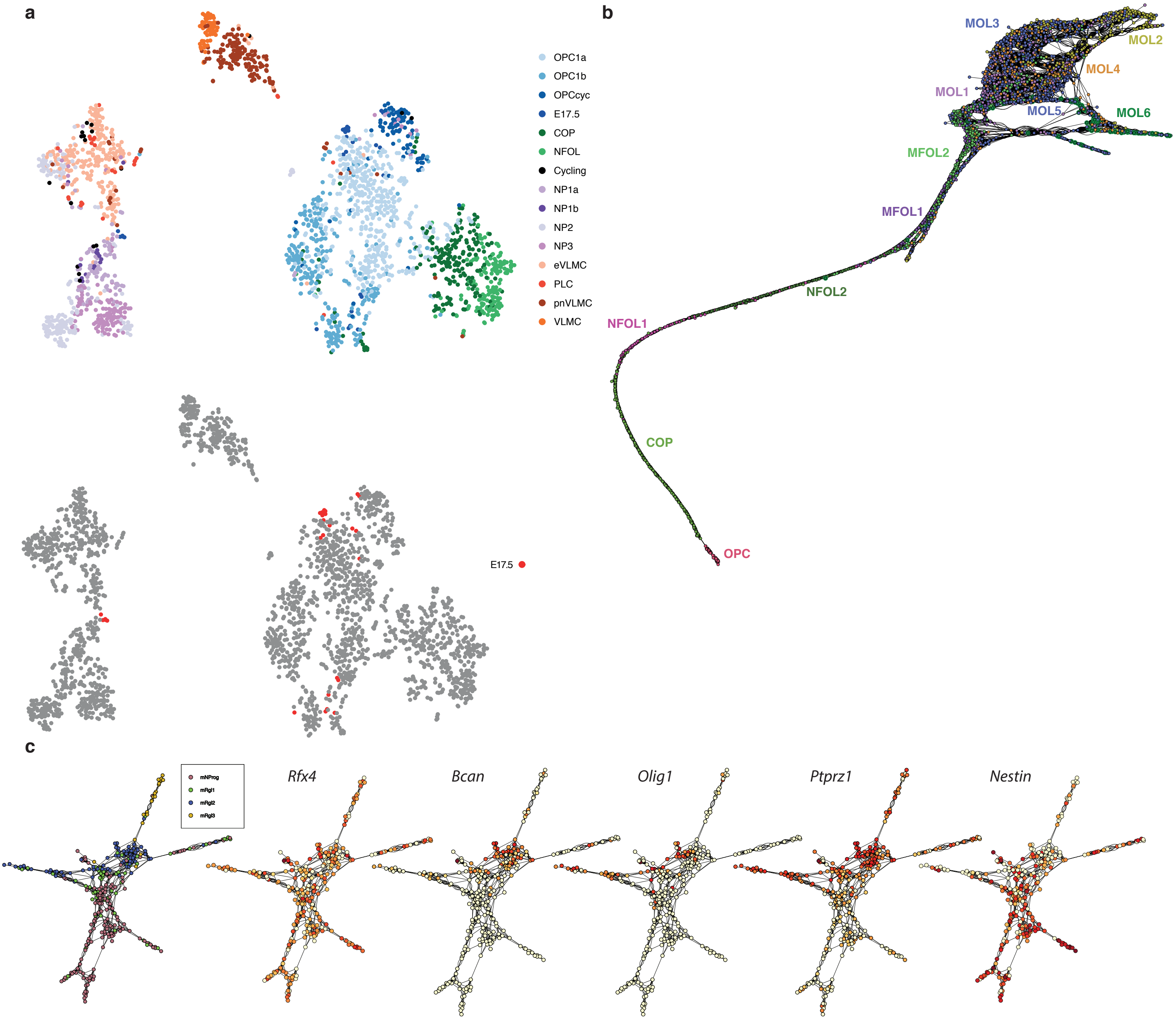

Figure S5 (related to Figure 3)

a) t-SNE representation of single cell data as in Figure 2c, including in addition E17.5 Pdgfra+/GFP cells

b) SCN3E network analysis of the 12 OL lineage populations identified in Marques et al 2016, as a proof of concept of the SCN3E; Myelin forming oligodendrocytes (MFOL)1 was suggested to be upstream mature oligodendrocytes MOL1, while MFOL2 was connected to MOL5;

c) SCN3E network analysis of embryonic ventral midbrain single-cell RNA-Seq from La Manno et all, 2016, with a focus on *Rfx4*+ populations

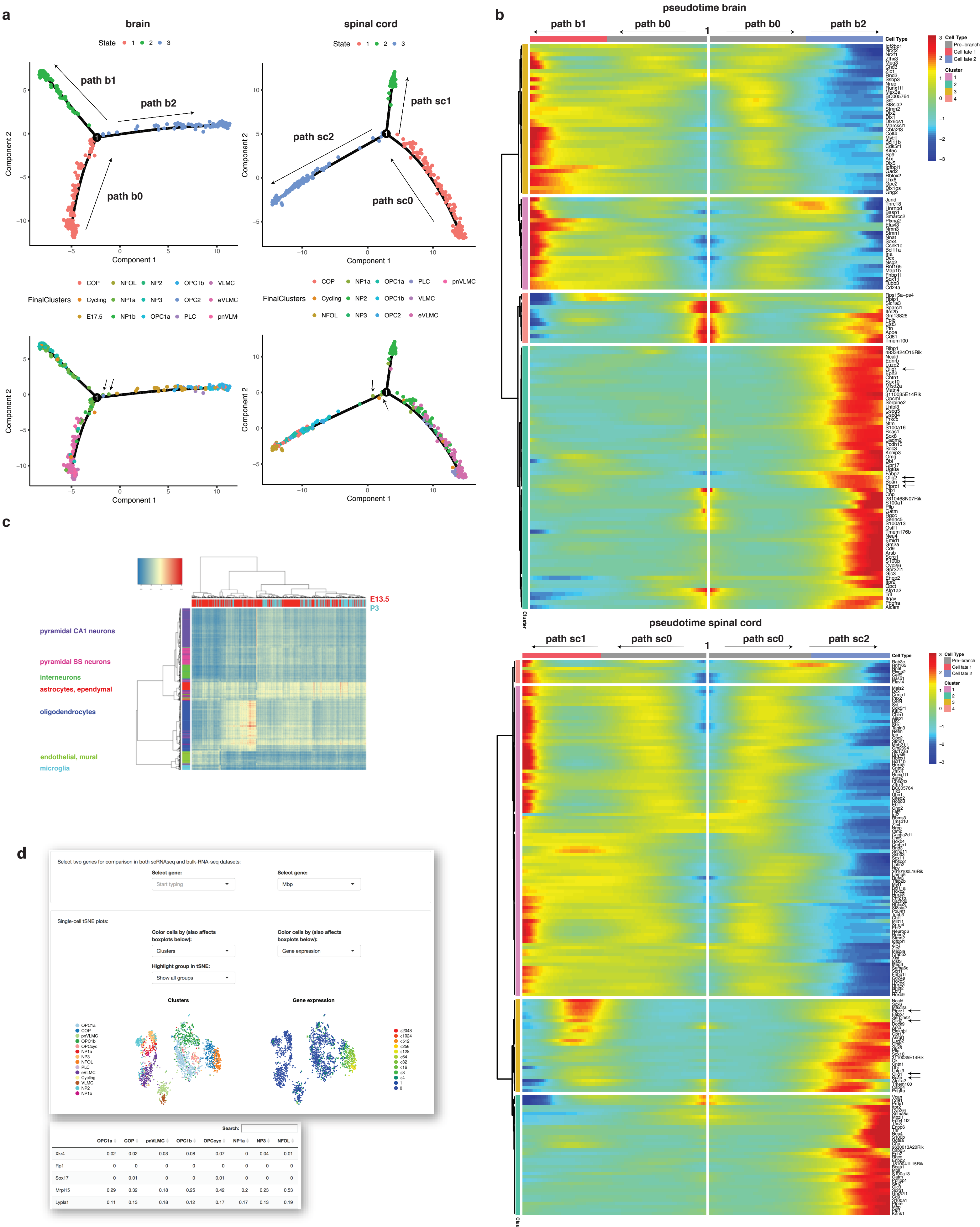

Figure S6 (related to Figures 3 and 4)

a) Reverse graph embedding deconvolved path depicting the captured differentiation trajectories in the single cell data from brain (left) and spinal cord (right) by Monocle2. Paths in the brain (b0/1/2) and spinal cord (s0/1/2) are shown as inferred by monocle, where the numbers 0, 1, and 2 stand for VLMC-, Neuronal-, and Oligodendrocyte- fates respectively. b) Differential expression heatmap capturing the most differentially expressed genes related to the oligodendrocyte (path 2) and neuronal lineage (path 1), with respect to the inferred branches in Monocle2. c) Pearson correlation performed on P7/8 single-cell RNAseq dataset obtained from a lineage tracing experiment with Pdgfra-Cre-ERT/RCE mice upon injection of tamoxifen at E12.5/13.5 and P3/P4 and Zeisel et al., 2015 single-cell RNA-Seq dataset. d) Screenshots of the resource website app, illustrating the exploration of expression data and table browsing.
